# Oral contraceptives containing ethinyl estradiol and drospirenone increase hydroxylation and methylation of endogenous estrogen but not genotoxic estrogen DNA-adduct formation

**DOI:** 10.1038/s41598-025-16892-8

**Published:** 2025-08-26

**Authors:** Gerda Venter, Carien L. van der Berg, Tarien Jacobs, Francois H. van der Westhuizen, Elardus Erasmus

**Affiliations:** 1 Biomedical and Molecular Metabolism Research, Faculty of Natural and Agricultural Sciences, North-West University (Potchefstroom Campus), Potchefstroom, South Africa; 2 Department of Biochemistry, Faculty of Natural and Agricultural Sciences, North-West University (Mahikeng Campus), Mmabatho, South Africa

**Keywords:** Combined oral contraceptives, Ethinyl estradiol, Drospirenone, Estrogen biotransformation, Catechol estrogens, Methylation, Estrogen DNA-adducts, Metabolomics, Hormones, Steroid hormones, Biochemistry

## Abstract

Combined oral contraceptives (COCs) are commonly prescribed for the prevention of pregnancy, as well as numerous other non-contraceptive health reasons. COCs act by suppressing the natural hormonal fluctuations of the menstrual cycle that result in ovulation. No studies have investigated the effects of COC use on endogenous estrogen biotransformation and the production of estrogen metabolites. This is important since imbalances in hormone biotransformation (e.g., inefficient methylation by catechol *O*-methyltransferases; COMT) are implicated in the initiation of breast cancer through the generation of genotoxic metabolites (i.e., estrogen quinones) and reactive oxygen species (ROS), and the depletion of vital antioxidants and metabolic cofactors. Here, we quantified the urinary levels of various estrogen precursors and metabolites in healthy young women who were using COCs containing drospirenone (DRSP) and ethinyl estradiol (EE) (*n* = 24) and controls (*n* = 25) via liquid chromatography–tandem mass spectrometry (LC‒MS/MS). In addition, we analysed several circulatory intermediates of the methylation cycle that are linked to the methylation of catechol estrogens via LC‒MS/MS. We found that free urinary estradiol (E2) and estrone (E1) were significantly lower, while 2-methoxyestrone (2-MeOE1) levels were significantly higher in COC users. Excretion of some metabolites including 16-hydroxylation pathway metabolites, glutathione conjugates, and DNA-adducts were also lower in COC users, although total hormone and metabolite excretion levels were not significantly different. Estrone metabolite ratios were higher in COC users, including 2&4-MeOE1:E1, 2&4-OHE1:E1, E1-3-sulphate: E1, and E1-3-glucuronide: E1. There was a positive correlation between 2-hydroxyestrogen and 2-methoxyestrogen levels in controls but not in COC users. In addition, the serum betaine and dimethylglycine (DMG) levels, as well as the betaine: choline ratio, were reduced in COC users, whereas the levels of choline and serine and the DMG: betaine ratio were significantly increased. DMG levels positively correlated with methoxyestrogens and methoxyestrogen: hydroxyestrogen ratios in COC users, while S-adenosylmethionine (SAM) negatively correlated with 2-MeOE2. Our data suggests that the use of EE/DRSP increases the flux of endogenous hormones into the hormone biotransformation pathway, resulting in increased conversion of estrogens (especially E1) into conjugated, catechol, and methylated estrogens but that the latter is limited by methyl-group donor availability. Interestingly, the increased oxidation of estrogens in COC users does not result in increased DNA-adduct formation.

**Supplementary Information:**

The online version contains supplementary material available at 10.1038/s41598-025-16892-8.

## Background

Contraceptive pills are commonly prescribed for the prevention of pregnancy, as well as numerous other non-contraceptive health reasons^1^. Various combinations of synthetic hormones are used in oral contraceptive formulations. Most of these formulations contain ethinyl estradiol (EE) as the synthetic estrogen in combination with various progestins. Combined oral contraceptives (COCs) containing drospirenone (DRSP) in combination with EE are classified as fourth-generation COCs and are generally regarded as milder in terms of their hormonal effects^2^.

COCs act by suppressing the natural hormonal fluctuations of the menstrual cycle that result in ovulation^3^. The hormonal (menstrual) cycle in women consists of the follicular, ovulatory, and luteal phases. During the follicular phase, 1–7 days following menstrual bleeding, estrogen concentrations are the lowest (~ 30 pg/ml). At days 12–14, estrogen concentrations peak at ~ 160 pg/ml just prior to ovulation, after which the concentrations in the luteal phase decline to ~ 40–100 pg/ml^4,5^. Various studies have reported that serum estrogen concentrations are within the physiological range of 30–160 pg/ml in premenopausal women^6,7^. However, it is worth noting that these values are only approximations because of the great variability among women, as well as within women across cycles. Research has shown that COC use, irrespective of brand or type, results in circulating endogenous estrogen concentrations similar to those of the early follicular phase of the menstrual cycle (20–50 pg/ml), although some formulations are associated with levels as high as 95–100 pg/ml^6,8,9^. This is because E2 production by the follicles in the ovaries is inhibited due to the inhibitory effect of the COC on follicle stimulating hormone (FSH) production, which normally results in follicular maturation.

Although these studies suggest that COC use suppresses ovarian E2 production, no studies have investigated the effects of its use on endogenous estrogen biotransformation and the production of estrogen metabolites in women. This is important since imbalances in hormone biotransformation are implicated in the initiation of breast cancer through the generation of genotoxic metabolites (i.e., estrogen quinones) and reactive oxygen species (ROS), and the depletion of vital antioxidants and metabolic cofactors^10,11^. Estrogen undergoes phase I and phase II biotransformation^12^ via a pathway that consists of different branches. In phase I reactions, CYP1A1/2 and CYP3A4 oxidise estrogen to a 2-hydroxylated catechol estrogen, whereas CYP1B1 (which is constitutively expressed in the breasts, ovaries, adrenal glands, and uterus, as well as in several other tissues^13,14^ converts it to 4-hydroxylated catechol estrogen^15,16^. Phase II estrogen conjugation includes sulfation and glucuronidation by sulfotransferases (SULTs) and UDP-glucuronosyltransferases (UGTs), respectively, as well as methylation by catechol *O*-methyltransferases (COMTs) and estrogen quinone glutathione (GSH) conjugation by glutathione-S-transferases^12^. The carcinogenic properties of estrogen are partly attributed to the production of quinones and semiquinones from 2- and 4-hydroxyestrogens that form depurinating DNA adducts and cause double-strand breaks in DNA^17^. Efficient methylation of catechol estrogens is, therefore, of foremost importance: during methylation, a methyl group is donated by S-adenosylmethionine (SAM) via COMT to ensure that catechol estrogens are converted to methoxyestrogens, thereby limiting the formation of genotoxic estrogen quinones^18^.

We and others have shown that COCs containing EE inhibit CYP1A2-mediated caffeine^19,20^ and tizanidine^21^ clearance. EE has also been reported to inhibit other CYP enzymes (including CYP3A4), as well as sulfatases (SULTs) and glucuronidases (UGTs)^22^. In vitro studies in rats indicated that oral EE treatment resulted in reduced uterine *Cyp1a1* mRNA expression, as well as intestinal *Cyp1a1*, *Nqo1*, and *Gsta2* expression^23^ whereas Hultman et al.^24^ reported that EE differentially affected various biotransformation genes (including *Cyp*, *Comt*, *Sult*, and *Gst*) in hepatocytes of rainbow trout. Here, we analysed and compared the urinary estrogen metabolite and precursor profiles of premenopausal women using COCs containing EE and DRSP with those of nonusers to determine how COC use affects endogenous estrogen biotransformation homeostasis. In addition, we analysed several circulatory intermediates of the methylation cycle (including the methionine‒homocysteine, folate, and transsulfuration pathways), which are linked to the methylation of catechol estrogens by COMT.

## Materials and methods

### Recruitment of participants and sample collection

The aim of this cross-sectional study was to analyse and compare the endogenous urinary estrogen metabolite profiles of healthy young women who were either long-term chronic users of COCs containing DRSP and EE or who were not using any hormonal products. Participants between 18 and 35 years of age were recruited between April 2017 and October 2019 from the general public by distributing printed flyers within the Potchefstroom urban areas and by using the social media platforms of the North-West University (NWU). The exclusion criteria were BMI ≥ 30, HIV-positive status, irregular menstrual cycle, pregnancy or breastfeeding in the past 6 months, COC/hormone use in the past 4 years (controls), use of COC formulations other than DRSP/EE, use of COC for less than 3 months, self-reported indications of diabetes, liver or kidney disease, asthma, other chronic diseases, chronic medication use, and smoking more than 5 cigarettes/day. Power calculations indicated that a minimum sample size of 20 was needed. In total, 49 eligible Caucasian women were included in the study (25 controls, 24 COC users). The COC used contained either 3 mg of DRSP and 0.03 mg of EE (*n* = 9) or 3 mg of DRSP and 0.02 mg of EE (*n* = 16).

All participants underwent HIV testing at the Student Counselling and Development division of the NWU Health Care Centre. Sample collection was performed in the luteal phase of the menstrual cycle. Since cycle length varies between women, individual sampling dates were calculated as described in Venter et al.^20^. First morning urine samples were collected by the participants at home and brought to the NWU before 10:00 am, where it was stored at −20 °C until analysis. On the same day, venous blood samples were drawn by a medical professional into BD Vacutainer™ Venous Blood Collection Tubes (SST™) via antecubital venepuncture, and the serum was stored at −80 °C until analysis.

### Reagents

Internal standards in the form of d4-labelled stable isotopes of estrone (E1), estradiol (E2), and estriol (E3), and d9-labelled isotope of progesterone were purchased from Steraloids (Newport, RI). Isotopes of 2- and 4-OHE1 for use as internal standards were obtained from Toronto Research Chemicals (North York, Canada). Dansyl chloride, sodium bicarbonate, ammonium formate and 2-acetamidophenol (used as internal standard) were purchased from Sigma Aldrich. All Burdick and Jackson Brand® solvents and water used in the liquid chromatography (LC)–tandem mass spectrometry (MS/MS) instrument were obtained from Honeywell Research Chemicals (Bucharest, Romania).

### Urine sample preparation

For parent hormone and estrogen metabolite analyses, the urine samples were diluted 1:5 with water. After dilution, an estrogen isotope and internal standard mixture with a final concentration of 50 ng/ml were added to the diluted urine sample. The diluted urine sample was then centrifuged at 900 × g for 10 min, and the supernatant was used for further analysis. The Strata C18-E (500 mg/3 ml) solid phase extraction (SPE) columns were sequentially conditioned with 6 ml of acetone, 6 ml of methanol (MeOH), and 6 ml of distilled water. The prepared urine sample supernatant (pH 7) was then loaded onto SPE columns. Each column was then washed with 6 ml of 5% (v/v) MeOH in water followed by 6 ml of 30% (v/v) MeOH in water, from which the sulphate and glucuronide conjugates were collected. The SPE cartridge was then washed again with 45% (v/v) MeOH in water solution and dried under vacuum for 5 min. The more nonpolar metabolites were subsequently eluted with 2 ml of MeOH and 2 × 2 ml of acetone. The SPE eluates were then evaporated to dryness either by freeze drying or under nitrogen gas. To the first (more polar), dried eluent, 100 µl of water and 100 µl of MeOH were added to yield a final volume of 200 µl. The resuspended samples were filtered through 0.2 μm nylon Spin-X® filters and transferred to glass LC analysis inserts in vials. The second (dried) eluent of nonpolar metabolites underwent dansylation derivatisation.

For extraction of DNA adducts, Strata X SPE cartridges (200 mg/6 ml) were conditioned with 12 ml of acetone followed by 12 ml of methanol and equilibrated with 12 ml of distilled water. The defrosted sample aliquots were centrifuged at 900 × g. The supernatant (1 ml) was diluted 4 times with water, and 1 ml of diluted internal standard was added for a final E2-d4 concentration of 50 ng/ml. The prepared sample (6 ml) was then loaded onto equilibrated SPE cartridges and allowed to flow through under gravitation. This was followed by washing with 12 ml of premixed 30% (v/v) methanol in water, and the cartridges were allowed to dry under vacuum for 5 min. The compounds of interest were eluted with 12 ml of one-part methanol (4 ml) and two parts acetone (8 ml). The eluates were collected in one tube. The samples were dried under a gentle stream of nitrogen gas until completely dry before being derivatised.

### Derivatisation

Sodium bicarbonate buffer (100 mM) was prepared in water, and different dansyl chloride solutions (1 mg/ml and 2 mg/ml) were prepared in acetone. To the more nonpolar dried eluents, 50 µl of dansyl chloride (1 mg/ml) and 50 µl of sodium bicarbonate buffer were added, and the mixture was incubated at 60 °C for 5 min. The dried DNA adduct eluents were dansylated with 50 µl of dansyl chloride (2 mg/ml) and 50 µl of sodium bicarbonate buffer and incubated at 50 °C for 5 min. After the incubation period, the samples were left to cool before being diluted 1:1 with methanol for a final sample volume of 200 µl. Dansylated samples were filtered through 0.2 μm nylon Spin-X® filters for LC, ensuring that no particulate matter entered the LC column. The filtered sample was transferred to glass LC analysis inserts in vials.

### Liquid chromatography‒mass spectrometry analyses of urinary estrogen metabolites

The prepared samples containing estrogen metabolites were analysed via separate methods as described by van der Berg et al.^25^ but with some modifications. For all the methods, 10 µl of the resuspended samples were injected onto an Agilent Technologies Zorbax eclipse plus an RRHD C8-E chromatographic column (2.1 mm × 100 mm, 1.8 μm) at a controlled temperature of 40 °C. Mobile phase A consisted of 95% water, 5% acetonitrile with 5 mM ammonium formate and 0.1% formic acid, and mobile phase B consisted of 100% acetonitrile with 0.1% formic acid. For all the methods, the mobile phase flow rate was maintained at 0.4 ml/min. Analytical analysis was performed on an Agilent Technologies 1200 series LC and 6460 electrospray ionisation (ESI) instrument with a Jetstream tandem mass spectrometer (MS). For analysis of the more polar metabolites, the mobile phase gradient started at 0% mobile phase B and increased to 20% in 0.5 min. At 20%, it was further increased to 22% in 4.5 min. The percentage mobile phase B was then increased to a maximum of 100% in 0.5 min and kept at 100% for 4.5 min before it was reduced to 0% B in 1 min. The postrun time was set at 5 min for a final run time of 15 min. A gas temperature of 250 °C, a gas flow of 10 l/min, a nebuliser pressure of 30 psi, a sheath gas temperature of 400 °C, a 12 l/min sheath gas flow, a capillary voltage of 3800 V for negative ionisation and a nozzle voltage of 500 V for negative ionisation were used. For the nonpolar metabolites, the mobile phase gradient started at 20% B and increased to 40% in 0.5 min. At 40%, it was further increased to 54% in 5.5 min, where it was held at 54% for 1 min. Then, the percentage B was increased to 55% in 5 min and 67% in 6 min, whereafter it was increased to 70% in 4 min. The percentage mobile phase B was then increased to 95% in 1 min, and to 98% in another 2 min. Mobile phase B was then held at 98% for 1 min, after which it was increased to a maximum of 100% and maintained at 0.5 min before being reduced back to 20% mobile phase B in 0.5 min. The postrun time was set at 5 min for a final run time of 35 min. A gas temperature of 250 °C, a gas flow of 8 l/min, a nebuliser at 20 psi, a sheath gas of 300 °C, a 10 l/min sheath gas flow, a capillary voltage of 1600 V for positive ionisation, and a nozzle voltage of 500 V were used. Multiple reaction monitoring (MRM) was conducted with specific transitions, collision and fragmentor energies, and retention times for each metabolite^25^.

For DNA-adduct analysis, the chromatographic gradient started at 10% B and increased to 50% in 0.5 min. From 50%, it was further increased to 70% in 4.5 min. The percentage of mobile phase B was then increased to 100% mobile phase B in 1 min and kept isocratic at 100% for 6 min before it was reduced to 10% B in 1 min. The postrun time was set at 5 min, for a final run time of 18 min. The mass spectrometry source settings were as follows: gas temperature of 350 °C, gas flow rate of 8 l/min, nebuliser pressure of 20 psi, capillary voltage of 1600 V and nozzle voltage of 500 V. For the Jet stream, the sheath gas temperature was set to 300 °C, with a 12 l/min sheath gas flow. The dansylated DNA adducts and dansylated stable isotope of E2 were all detected via positive ionisation in MRM mode, with specific precursor/product ion transitions identified and with optimal fragmentor and collision energies^26^.

The dansylated DNA-adduct samples were also used for the analysis of estrogen GSH conjugates. For this analysis, the mobile phase gradient started at 20% and was maintained for 1 min. Mobile phase B increased to 70% in 2 min. From 70%, it was further increased to 100% in 3 min and held at 100% for 4 min before it was reduced to 20% B in 1 min. The postrun time was set at 4 min for a final run time of 15 min. A gas temperature of 250 °C, gas flow of 8 l/min, nebuliser at 20 psi, sheath gas temperature of 300 °C, with a 10 l/min sheath gas flow, a capillary voltage of 1600 V for positive ionisation, and a nozzle voltage of 500 V were used. The MRM transitions for the dansylated GSH conjugates and their metabolic products for the 2-hydroxyestrogens were as follows: 2OHE1-1/4-N-accetylcysteine (NAcCys), 681.2->171.1; 2-OHE1-1/4-glutathione (SG), 825.0->171.1; 2-OHE1-1/4-cysteine (Cys), 639.2->171.1; 2-OHE2-1/4-NAcCys, 683.2-> 171.1; 2-OHE2-1/4-SG, 827.7->171.1; and 2-OHE2-Cys, 641.2->171.1. The 4-hydroxyestrogen SG, NacCys, and Cys conjugates had the same MRM transitions as the 2-hydroxyestrogens did, and these metabolites were, therefore, separated only by chromatographic retention. E2-d4 was used as an internal standard for this method, with the transition 510.0->171.1.

### Sample preparation and LC‒MS/MS analyses of serum methylation cycle metabolites

The procedures followed for sample preparation and analyses of serum methylation cycle metabolites are described in the Supplementary file.

### Osmolality measurements

The osmolality of all the urine samples was measured at Ampath Laboratories, South Africa, by determining the freezing-point of each sample using an Advanced Osmometer (model 3320).

### Data processing and statistical analysis

The LC‒MS/MS data were processed via MassHunter Quantitative Analysis software (version B.06.00, build 6.0.388.0) to quantify the individual metabolite levels relative to the stable isotopes added^25,26^. The urinary metabolite concentrations (in ng/l) were normalised to osmolality (mOsmol/kg). The normalised concentrations of individual metabolites were used to calculate certain metabolite ratios and to calculate the fractions of all the metabolites analysed. To identify potential outliers, principal component analysis was performed after log transformation and Pareto scaling were applied to the entire dataset using MetaboAnalyst 5.0 online software (https://www.metaboanalyst.ca/MetaboAnalyst/home.xhtml). Two control samples were identified as outliers and removed from the dataset before further statistical analyses were performed. Univariate analysis was performed on the untransformed data to identify statistically significant differences between the control and COC user groups. The datasets were complete, i.e., there were no missing data. Nonparametric univariate analyses were performed using IBM SPSS Statistics (version 30.0.0.0; https://www.ibm.com/spss) and included the Mann‒Whitney test and calculation of its associated effect size (ES) via Microsoft Excel. Differences between groups were considered to be of practical significance when the ES was ≥ 0.3. Correction for multiple testing was performed via the two-stage linear step-up procedure of Benjamini, Krieger and Yekutieli available in GraphPad Prism 10 (https://www.graphpad.com/) to control the rate of false discovery (BKY-FDR). A false discovery rate of 5% was considered acceptable. Therefore, an adjusted *p* value ≤ 0.05 was considered statistically significant. GraphPad Prism 10 was also used to construct graphs and perform Spearman correlation analysis.

## Results

### Urinary free estrogen levels are reduced in COC users although total hormone excretion is not

First, the urinary concentrations of individual estrogen metabolites were compared between controls and COC users. Osmolality was chosen as the parameter for normalisation, since the creatine concentrations differed between the two groups, while osmolality was much more consistent (Supplementary Fig. S1). Among the 39 variables, nine had significantly lower concentrations in the COC users with ES ≥ 0.3 (Table 1) and included the two parent estrogens (E1 and 17β-estradiol), as well as estriol-3-sulphate (E3-3-sulphate), 2&4-hydroxyestradiol-cysteine (2&4-OHE2-Cys), 2&4-hydroxyestrone-glutathione (2&4-OHE1-SG), 4-hydroxyestradiol-1-N7-guanine (4OHE2-1-N7Gua), 16-epiestriol, 16-keto-estradiol, and 2&4-hydroxyestradiol-glutathione (2&4-OHE2-SG). The variable with the greatest ES was E1, whose urine concentration was about four times greater in the control women than in the COC users. Only one variable was significantly higher in the COC group, namely 2-methoxyestrone ([2-MeOE1; ES = 0.33). Interestingly, although most of the metabolites tended to be lower, six metabolites tended to be higher in the COC group including all four catechol estrogens (i.e. 2-hydroxyestrone [2-OHE1], 2-hydroxyestradiol [2-OHE2]), 4-hydroxyestrone [4-OHE1], and 4-hydroxyestradiol [4-OHE2]), as well as E1-3-glucuronide and 2&4-hydroxyestrone-N-acetyl-cysteine (2&4-OHE1-NAcCys). This subtle elevation in catechol estrogen and reduction in other metabolite levels was also observed when metabolite groups were compared (Table 2), although these effects were not of practical significance (ES < 0.3). Interestingly, despite the significant reduction in total parent estrogen (E2 + E1) levels in the COC users, the total hormone and metabolite excretion levels were not lower.

**Table 1:** Individual urinary parent estrogen, precursor, and metabolite levels.

Variable (ng/l normalised to mOsmol/kg))	Control (*n* = 23)				COC (*n* = 24)				Mann–Whitney effect size	BKY-FDR *p* value
	Min	Max	Mean	Std. dev.	Min	Max	Mean	Std. dev.		
E1	24.86	989.02	**217.04**	251.66	2.42	585.53	**51.92**	116.34	0.66	< 0.001
E3-3-sulphate	6.05	2161.10	**171.94**	448.17	0.14	647.32	**51.49**	142.67	0.56	0.004
2&4-OHE2-Cys	59.48	1426.36	**590.27**	337.12	30.67	1628.56	**336.92**	415.88	0.49	0.016
2&4-OHE1-SG	10.47	1099.87	**210.65**	257.05	5.98	283.66	**65.85**	72.02	0.44	0.046
17β-Estradiol	0.14	17.56	**3.04**	4.04	0.05	16.45	**1.87**	3.79	0.37	0.103
4OHE2-1-N7Gua	10.95	3673.90	**557.75**	874.94	6.02	1452.50	**194.28**	377.63	0.36	0.103
2-MeOE1	0.06	26.66	**2.47**	5.56	0.03	20.52	**4.53**	5.03	0.33	0.158
16-Epiestriol	0.02	63.30	**7.53**	14.98	0.03	25.47	**2.08**	5.35	0.30	0.177
16-Ketoestradiol	0.19	44.99	**5.57**	10.00	0.03	34.98	**3.47**	7.37	0.30	0.177
2&4-OHE2-SG	15.75	438.50	**140.74**	134.77	1.69	1203.43	**106.80**	241.43	0.30	0.177
E3	0.24	65.56	**9.76**	15.80	0.26	41.26	**3.04**	8.20	0.29	0.182
2OHE1-6-N3Ade	6.58	423.79	**80.71**	96.12	4.69	215.53	**42.43**	48.33	0.29	0.196
Androstenedione	9.88	384.78	**49.11**	79.75	6.79	108.42	**22.70**	20.09	0.27	0.230
16-OHE1	0.12	55.18	**7.38**	14.75	0.06	34.86	**2.66**	7.06	0.27	0.240
4OHE1-1-N7Gua	4.91	6908.68	**1247.39**	1975.00	2.42	6368.28	**697.36**	1718.64	0.24	0.321
4-MeOE2	0.02	1.72	**0.25**	0.39	0.02	0.79	**0.15**	0.20	0.22	0.354
4OHE1-1-N3Ade	0.28	19.67	**3.88**	4.33	0.03	23.64	**3.35**	5.48	0.22	0.354
4OHE2-1-N3Ade	0.59	15.31	**3.69**	3.68	0.05	13.48	**2.97**	3.36	0.19	0.442
2&4-OHE2-NAcCys	17.36	7847.40	**1778.37**	2214.04	56.19	5749.44	**1188.86**	1579.30	0.19	0.444
E3-16-glucuronide	15.92	3768.41	**801.60**	933.88	23.34	873.07	**356.39**	218.69	0.16	0.513
2-MeOE2	0.03	1.84	**0.21**	0.37	0.01	0.93	**0.20**	0.28	0.15	0.539
4-MeOE1	0.11	200.51	**13.52**	41.58	0.21	100.44	**14.68**	28.36	0.15	0.551
Progesterone	2.79	106.56	**21.75**	21.89	0.68	70.90	**16.74**	15.52	0.14	0.566
2&4-OHE1-Cys	3.90	467.01	**58.20**	96.26	2.33	2030.01	**135.78**	412.87	0.14	0.581
2-OHE1	0.88	305.44	**79.14**	89.85	0.31	1029.49	**195.04**	299.56	0.12	0.669
E1-2-hydroxy-3-methyl ether	0.02	2.17	**0.40**	0.50	0.04	1.55	**0.30**	0.32	0.11	0.702
2-OHE2	2.25	1534.31	**369.01**	442.25	1.94	11443.39	**1602.83**	3056.16	0.11	0.716
2&4-OHE1-NAcCys	8.03	3993.73	**607.02**	913.25	2.95	3723.13	**579.89**	924.78	0.10	0.740
2OHE2-6-N3Ade	1.83	137.33	**18.39**	28.34	0.85	150.09	**16.50**	30.18	0.07	0.840
E2-3-sulphate	0.17	1254.48	**81.43**	259.25	0.41	149.94	**29.54**	38.44	0.06	0.840
4-OHE2	0.21	141.72	**27.17**	31.64	0.05	344.77	**44.77**	83.60	0.04	0.882
E2-17-sulphate	0.05	71.02	**6.05**	14.85	0.07	67.21	**4.62**	13.47	0.04	0.882
E1-3-sulphate	0.49	678.96	**61.50**	145.85	0.54	138.46	**31.47**	41.76	0.04	0.882
4-OHE1	0.59	368.50	**49.81**	75.75	0.16	566.57	**73.71**	127.33	0.03	0.908
E1-3-glucuronide	66.68	54803.03	**5980.26**	12357.77	24.00	114400.65	**13838.28**	31092.25	0.02	0.933
Testosterone	1.20	480.19	**27.34**	98.98	0.63	66.89	**9.64**	13.95	0.01	0.964
17α-Estradiol	0.17	7.43	**1.46**	1.57	0.36	3.02	**1.24**	0.86	0.01	0.972
17-Epiestriol	0.10	301.13	**31.75**	71.72	0.10	121.37	**12.33**	25.70	0.00	0.972
E2-3-glucuronide	0.82	216.09	**36.41**	59.11	0.13	115.36	**22.68**	27.39	0.00	0.972

Means are indicated in bold. *E2: estradiol*,* E1: estrone*,* E3: estriol*,* MeO: methoxy*,* OH: hydroxy. ES r < 0.3: small effect; between 0.3 and 0.5: medium effect; > 0.5: large effect.*

**Table 2:** Total urinary estrogen metabolite levels.

Variable (ng/l normalised to mOsmol/kg))	Control (*n* = 23)				COC (*n* = 24)				Mann‒Whitney effect size	BKY-FDR *p* value
	Min	Max	Mean	Std. dev.	Min	Max	Mean	Std. dev.		
Total estrogens (E2 + E1)	28.08	1009.73	**221.54**	256.01	2.85	602.75	**55.03**	119.38	0.66	< 0.001
2-Methoxy estrogens (including E1-2OH-3-methyl ether)	0.20	30.01	**3.07**	6.17	0.16	21.15	**5.03**	5.36	0.31	0.177
Total 4-OH DNA adducts	33.59	8592.89	**1812.71**	2402.45	8.51	7821.23	**897.96**	1989.24	0.28	0.205
Total 16-OH metabolites (including E3)	1.41	493.78	**61.99**	110.38	0.80	241.80	**23.58**	49.25	0.23	0.349
Total 2-OH DNA adducts	8.41	561.12	**99.09**	119.78	5.66	238.90	**58.93**	59.56	0.23	0.349
Total GSH Conjugates	503.24	12988.58	**3385.24**	3090.44	217.57	9096.74	**2414.11**	2416.66	0.20	0.377
4-Methoxy estrogens	0.18	200.67	**13.77**	41.62	0.24	100.91	**14.83**	28.39	0.18	0.449
Total E3 (parent + metabolites)	23.68	4086.60	**996.40**	1171.88	40.76	1364.69	**416.46**	318.76	0.18	0.449
2-OH estrogens	3.12	1810.79	**448.15**	512.09	2.26	11701.62	**1797.87**	3110.89	0.15	0.562
Total E2 (parent + metabolites)	778.67	11668.12	**3614.23**	2689.45	357.18	13610.44	**3554.23**	3318.16	0.10	0.724
Total E1 (parent + metabolites)	1495.13	55663.54	**8650.70**	11967.05	801.57	115783.68	**15749.28**	31275.00	0.07	0.824
TOTAL (all hormones + metabolites)	2703.39	58008.23	**13359.93**	12086.15	2108.36	118064.75	**19769.37**	30666.89	0.07	0.824
4-OH estrogens	0.79	510.22	**76.97**	104.00	0.21	568.95	**118.48**	148.95	0.04	0.882
Total sulphate and glucuronide conjugates	90.40	55122.43	**7139.19**	12794.29	169.96	115287.75	**14334.47**	31145.51	0.02	0.922

Means are indicated in bold. *Estrogen metabolites were grouped according to the type or branch of the estrogen biotransformation pathway*,* and the levels of the individual metabolites in the group were combined. TOTAL (all hormones + metabolites) also includes estrogen precursor hormones. E2: estradiol*,* E1: estrone. Estrogen: estradiol and estrone. ES r < 0.3: small effect; between 0.3 and 0.5: medium effect; > 0.5: large effect.*

### COC use increases the hydroxylation, methylation, and conjugation of estrone

Other studies^7–9^ have shown that the use of synthetic hormones reduces circulatory progesterone and E2 levels. Here, we also observed reduced urinary levels of the endogenous parent estrogens (E2 and E1) in COC users (Table 1). However, total hormone metabolite levels were not changed but in fact showed a tendency to be higher in the COC users (Table 2). This may suggest that the reduction in free estrogen levels is a result of increased sulphate and glucuronide conjugation or increased oxidation via the estrogen biotransformation pathway. Therefore, to determine whether the flux through the hormone biotransformation pathway was affected by COC use, we calculated certain metabolite ratios (Table 3). This included metabolite: parent ratios, as well as metabolite: metabolite ratios. Forty-five hormone metabolite ratios were calculated, 12 of which were significantly greater in the COC users than in the controls (ES ≥ 0.3). Among these, 11 were ratios between E1 and its metabolites, which included methylated and hydroxylated E1, as well as some 16-OH pathway metabolites, E1 DNA-adducts, and E1 sulphate, glucuronide, and GSH conjugates. Of note was the large (8- to 13-fold) increase in the conversion of E1 into its hydroxylated and methylated forms compared to the 2- to 5-fold increase observed for most other metabolite ratios. To get an indication of how efficiently the catechol estrogens were methylated we also determined the methylated : hydroxylated estrogen ratios. Interestingly, except for the 4MeOE2 : 4OHE2 ratio, all other methylation ratios were not increased to the same extent as the hydroxylation ratios. Finally, there was a significant increase in the E1-3-sulphate : E1, E1-3-glucuronide : E1, and the total sulphate and glucuronate conjugated estrogens to free estrogens (Conjugated: Free estrogens) ratio.

**Table 3:** Estrogen metabolite ratios.

Ratio	Control (*n* = 23)				COC (*n* = 24)				Mann‒Whitney effect size	BKY-FDR *p* value
	Min	Max	Mean	Std. dev.	Min	Max	Mean	Std. dev.		
2-MeOE1 : E1	0.00	0.22	**0.02**	0.05	0.00	0.94	**0.18**	0.21	0.65	< 0.001
2&4-OHE1-Cys : E1	0.01	9.21	**0.77**	1.87	0.02	109.80	**7.50**	22.36	0.47	0.026
4OHE1-1-N3Ade : E1	0.00	0.33	**0.04**	0.07	0.00	0.53	**0.11**	0.14	0.42	0.052
2-Hydroxyestrone : E1	0.00	11.12	**1.27**	2.67	0.05	70.77	**13.44**	23.37	0.42	0.054
17-Epiestriol : E1	0.00	2.19	**0.31**	0.66	0.01	7.64	**0.78**	1.95	0.40	0.066
Conjugated : free estrogens	0.45	565.88	**58.83**	118.26	2.12	5370.17	**607.25**	1406.97	0.38	0.081
4-OHE1 : E1	0.00	1.64	**0.46**	0.57	0.02	30.65	**4.09**	7.74	0.36	0.103
2OHE1-6-N3Ade : E1	0.01	15.08	**1.26**	3.09	0.05	9.87	**2.45**	3.10	0.36	0.103
E1-3-sulphate : E1	0.01	2.05	**0.39**	0.65	0.01	23.04	**2.33**	4.85	0.35	0.124
E1-3-glucuronide : E1	0.28	588.23	**54.28**	122.04	1.08	9636.34	**841.93**	2216.69	0.34	0.124
4-MeOE1 : E1	0.00	0.20	**0.04**	0.04	0.00	4.20	**0.55**	0.97	0.33	0.152
16-OHE1 : E1	0.00	0.40	**0.05**	0.10	0.00	1.87	**0.12**	0.37	0.31	0.177
Total GSH Conjugates : total estrogens	2.24	220.23	**37.22**	50.59	3.99	1380.15	**198.90**	347.69	0.29	0.182
2&4-OHE2-Cys : E2	18.47	1711.37	**261.75**	342.29	11.39	2691.49	**308.69**	642.87	0.28	0.200
DNA Adducts : Total estrogens (DNA adducts/Parent + hydroxy & methoxyestrogens)	0.10	27.86	**4.50**	6.41	0.01	97.02	**11.21**	25.40	0.26	0.240
2-Hydroxyestrogens : 16-Hydroxyestrogens	3.44	6959.35	**897.76**	1833.61	6.72	37479.52	**3331.73**	7965.24	0.26	0.257
2-OHE1 : 16-OHE1	0.43	1768.53	**167.61**	402.18	1.62	10753.88	**722.12**	2208.88	0.24	0.308
2&4-OHE1-NAcCys : E1	0.03	71.53	**6.47**	14.87	0.09	686.35	**82.67**	179.94	0.22	0.354
E3-16 glucuronide : E3	2.28	2995.94	**497.37**	868.57	2.02	1349.59	**427.84**	394.99	0.21	0.354
4OHE2-1-N7Gua : estradiol	4.15	1401.13	**188.43**	334.96	1.69	2454.07	**168.73**	499.38	0.21	0.354
E3-3-sulphate : E3	1.38	589.64	**50.96**	122.36	0.08	485.71	**36.47**	103.05	0.21	0.354
2-Hydroxyestrogens : 4-Hydroxyestrogens	1.53	38.52	**7.31**	7.80	1.61	280.49	**23.00**	56.40	0.20	0.385
2-OHE2 : E2	1.36	735.99	**131.67**	182.89	2.72	3923.70	**547.41**	986.01	0.19	0.444
16-OHE1 : total estrogens	0.00	0.12	**0.01**	0.03	0.00	0.04	**0.01**	0.01	0.18	0.447
2&4-OHE1-SG : E1	0.03	8.84	**1.93**	2.29	0.06	52.29	**5.82**	11.01	0.17	0.485
2-MeOE1 : 2-OHE1	0.00	0.59	**0.08**	0.15	0.00	2.71	**0.44**	0.85	0.16	0.537
2&4-OHE2-SG : E2	2.29	210.45	**48.36**	54.39	0.76	427.70	**56.87**	98.81	0.16	0.537
E2-3-sulphate : E2	0.06	637.21	**37.30**	131.48	0.02	69.17	**15.39**	18.09	0.16	0.537
4-MeOE2 : 4-OHE2	0.00	0.56	**0.08**	0.15	0.00	13.99	**0.68**	2.85	0.15	0.540
16-Epiestriol : E3	0.03	5.40	**0.86**	1.17	0.05	22.43	**1.49**	4.52	0.14	0.581
2&4-OHE2-NAcCys: E2	3.20	4553.41	**746.83**	1099.86	7.37	8811.92	**955.48**	1893.03	0.11	0.720
E2-3-glucuronide : E2	0.07	109.76	**14.62**	27.26	0.04	54.26	**12.58**	14.54	0.11	0.720
2-MeOE2 : E2	0.01	0.31	**0.07**	0.08	0.00	0.35	**0.09**	0.10	0.10	0.722
2-MeOE2 : 2-OHE2	0.00	0.05	**0.01**	0.01	0.00	0.11	**0.01**	0.02	0.08	0.822
2OHE2-6-N3Ade : E2	0.20	52.37	**7.92**	13.21	0.31	144.78	**10.77**	28.84	0.07	0.828
4-OHE2 : E2	0.02	30.75	**8.13**	8.40	0.09	135.80	**16.48**	29.56	0.07	0.832
4OHE2-1-N3Ade : E2	0.07	4.86	**1.36**	1.30	0.02	3.07	**1.06**	0.88	0.07	0.832
2-Methoxyestrogens : 2-hydroxyestrogens	0.00	0.12	**0.02**	0.03	0.00	1.06	**0.10**	0.23	0.06	0.840
4-Methoxyestrogens : 4-hydroxyestrogens	0.00	11.00	**0.75**	2.28	0.00	31.01	**2.80**	7.93	0.06	0.840
4OHE1-1-N7Gua : E1	0.04	66.32	**8.02**	14.59	0.02	423.72	**50.57**	114.79	0.05	0.852
4 & 16-Hydroxyestrogens : total estrogens	0.01	1.06	**0.26**	0.31	0.00	1.01	**0.20**	0.21	0.05	0.854
4-MeOE2 : E2	0.01	0.75	**0.09**	0.16	0.00	1.25	**0.11**	0.25	0.04	0.886
16-Ketoestradiol : E3	0.01	5.04	**1.29**	1.42	0.05	5.70	**1.42**	1.75	0.04	0.886
E2-17-sulphate: E2	0.00	22.57	**2.16**	4.85	0.02	15.91	**1.84**	3.46	0.03	0.897
4-MeOE1 : 4-OHE1	0.00	14.74	**1.11**	3.10	0.00	53.54	**4.61**	13.84	0.01	0.972

Means are indicated in bold. *Estrogen metabolite ratios were calculated between each estrogen metabolite and its parent estrogen (i.e.*,* estradiol*,* estrone*,* or estriol) and between methoxy- and hydroxyestrogens. E2: estradiol*,* E1: estrone*,* E3: estriol*,* MeO: methoxy*,* OH: hydroxy. Estrogen: estradiol and estrone. ES r < 0.3: small effect; between 0.3 and 0.5: medium effect; > 0.5: large effect.*

### COC use alters the relative abundance of hormone biotransformation metabolites

**Fig. 1:**
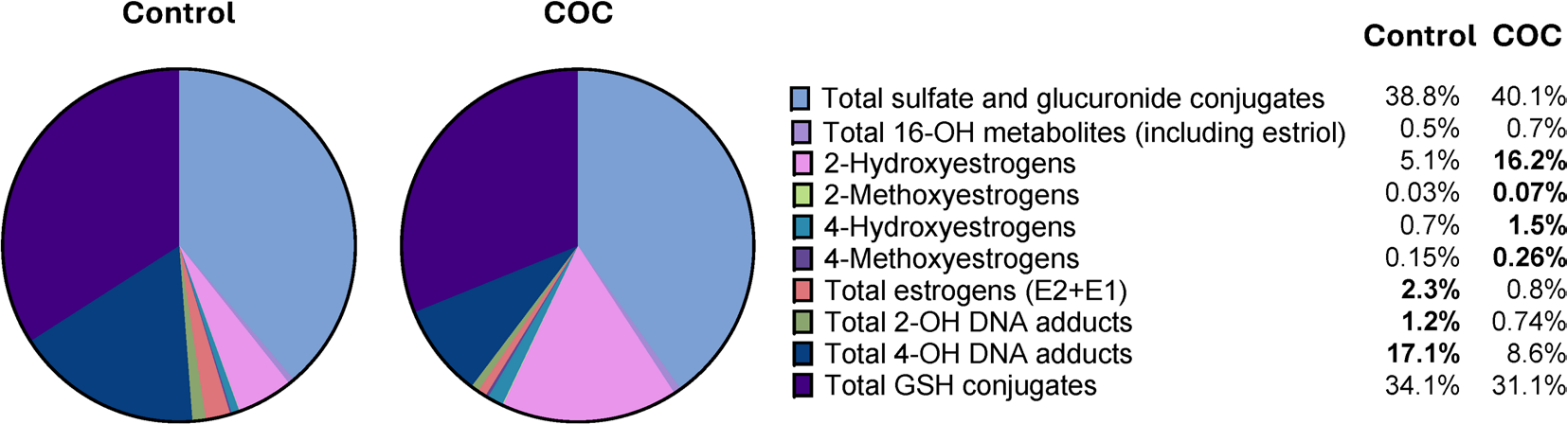
Estrogen metabolite abundances. Estrogen metabolites were grouped into different branches of the estrogen biotransformation pathway, and the abundances were calculated as a percentage of the total metabolite load (fraction of the whole). These percentages are indicated on the right-hand side of the figure. The total metabolite load was calculated as the sum of all parent estrogens and all estrogen metabolites and precursors. The percentages in bold indicate values that are > 1.5-fold greater than those of the other group.

As a further step in analysing the flux through the biotransformation pathway, we also calculated the metabolite fractions as a percentage of all the hormone analytes measured. In this way, it was possible to determine the most abundant urinary hormone metabolites in the control group and determine how their abundance was altered with the use of COCs. This was done for the individual metabolites (Supplementary Table S2), as well as for groups of metabolites, to determine which branch in the biotransformation pathway was altered in the COC users (Fig. 1). The results indicated that in both groups, the sulphate, glucuronide, and glutathione conjugates made up the largest fraction of the metabolites and were similar between the groups. The most abundant metabolite in both the control and COC user groups was E1-3-glucuronide, accounting for 28.55% and 34.02% of all the metabolites analysed, respectively (Supplementary Table S2). The 16-OH pathway metabolite abundance did not seem to be affected by COC use. However, a larger fraction of the estrogens was excreted as 2- and 4-hydroxylated and -methylated metabolites in the COC users than in the controls, with the largest shift visible for the 2-OH-estrogens (5.1% in controls vs. 16.2% in COC users; Fig. 1). Concurrently, the abundances of the 2- and 4-OH-estrogen DNA adducts were lower in the COC users, indicating that the increased formation of catechol estrogens did not result in increased adduct formation. Furthermore, in COC users, 2-OHE1 and 4-OHE1 were more abundant than E1 itself (Supplementary Table S2). For E1, the higher abundance of hydroxylated E1 also resulted in an increased abundance of 2- and 4-MeOE1, although these increases did not seem to be proportional to those observed for the hydroxylated metabolites. This was, however, not the case for the E2 metabolites: the abundance of 2- and 4-MeOE2 was still the lowest of all the metabolites and similar to that found in the control group (Supplementary Table S2).

### The maximum capacity to methylate 2-hydroxyestrone may be reached in COC users

**Fig. 2:**
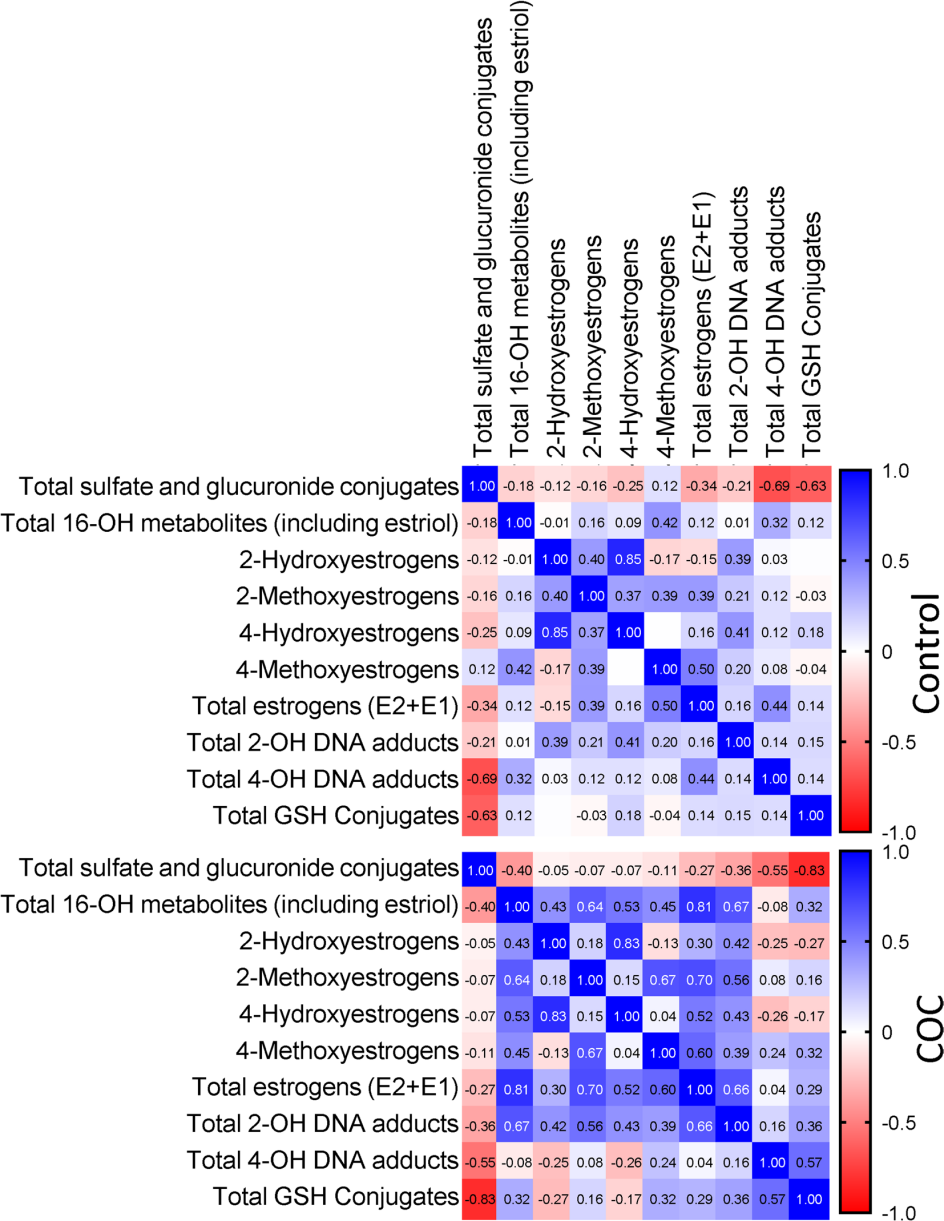
Spearman correlation between estrogen metabolite groups. Correlation analyses were performed for each experimental group separately using the grouped metabolite abundances calculated for Fig. 1. Spearman r values for each correlation are indicated in the individual squares of the heatmap. *P* values are given in Supplementary Tables S3 and S4.

Spearman correlation analysis between the different groups of metabolites revealed strong negative correlations between the total sulphate and glucuronide conjugates and the total glutathione conjugates (*r* = −0.63, *p* = 0.001 [Control]; *r* = −0.82, *p* < 0.0001 [COC]) as well as the total 4-OH DNA-adducts (*r* = −0.69, *p* < 0.001 [Control]; *r* = −0.55, *p* = 0.006 [COC]; Fig. 2 and Supplementary Tables S3 and S4). This means that the more estrogens are conjugated by sulphation or glucuronidation, the lower the conversion into estrogen quinones, which can subsequently be converted into glutathione conjugates or lead to the formation of DNA adducts. There was also a negative relationship between the total sulphate and glucuronide conjugates and the other metabolites, indicating that if the estrogens are not biotransformed, they are converted into sulphate or glucuronide conjugates, which agrees with the current understanding of estrogen biotransformation. A relatively strong (*r* = 0.4; *p* = 0.06) positive correlation existed between the levels of 2-hydroxyestrogens and 2-methoxyestrogens in the control group, indicating that increased 2-hydroxyestrogen levels result in increased methylation by COMT. The correlation between 4-hydroxyestrogens and 4-methoxyestrogens seemed to be much weaker (*r* = 0.003; *p* = 0.99). This may be due to the higher catalytic efficiency of COMT in the formation of 4-methylated estrogens than 2-methylated estrogens and the difference in the kinetics of the two reactions^27^. Interestingly, the strong positive correlation between 2-hydroxyestrogens and 2-methoxyestrogens was lost in the COC user group (*r* = 0.18; *p* = 0.41), while simultaneously the correlation between both these metabolites (but especially the 2-methoxyestrogens) and the 2-OH-DNA adducts became stronger and significant (2-hydroxyestrogens: *r* = 0.42; *p* = 0.04, and 2-methoxyestrogens: *r* = 0.55; *p* = 0.004). This supports the notion that methylation efficiency of the 2-hydroxyestrogens is under pressure.

### The levels of the methyl group donor betaine are reduced and its conversion into DMG increased in COC users

The activity of COMT and the efficiency of estrogen methylation depend on the availability of the methyl group donor SAM, which is obtained from the methionine‒homocysteine cycle. The methionine‒homocysteine cycle, in turn, obtains the methyl group from the folate cycle and supplies precursors for the production of glutathione and taurine via the transsulfuration pathway (Supplementary Fig. S2). To determine whether the limited upregulation of catechol estrogen methylation may be due to the limited availability of methyl group donors from these pathways, we analysed several intermediates from these pathways in the serum of the participants. These included L-homocysteic acid (HCA), taurine, dimethylglycine (DMG), serine, betaine, cysteine, glycine, riboflavin, homocysteine, choline, methionine, pyridoxine, 5-methyltetrahydrofolic acid (5-MTHFA), cystine, cystathionine, S-adenosylhomocysteine (SAH), homocystine, and SAM. In addition, the individual metabolite concentrations were used to calculate the ratios of betaine: choline and DMG: betaine, as well as total homocysteine (homocysteine and homocystine) and total cysteine (cysteine and cystine) contents.

Our results revealed that betaine and DMG levels, as well as the betaine: choline ratio, were reduced in COC users, whereas choline, serine, and the DMG: betaine ratio were significantly increased in COC users compared with controls (Table 4).

**Table 4:** Methylation cycle metabolite concentrations and ratios.

Variable (ng/ml)	Control (*n* = 23)				COC (*n* = 25)				Mann‒Whitney effect size	BKY-FDR *p* value
	Min	Max	Mean	Std. dev.	Min	Max	Mean	Std. dev.		
Betaine	1120.80	3865.11	2680.54	733.49	783.70	3245.09	1329.95	478.71	0.77	< 0.0001
Betaine: Choline	0.39	3.67	1.13	0.76	0.19	1.96	0.44	0.36	0.73	< 0.0001
DMG: Betaine	0.06	0.24	0.13	0.05	0.07	0.35	0.20	0.07	0.54	< 0.01
Choline	925.12	4221.18	2781.00	753.49	761.72	5189.12	3533.96	853.10	0.52	< 0.01
DMG	173.45	524.88	333.27	87.15	104.42	599.35	260.56	102.07	0.41	0.02
Serine	6437.93	15435.93	11245.89	1876.50	6961.22	16879.80	12655.11	2491.10	0.38	0.03
Methionine	7849.49	14811.40	11013.57	1900.21	8541.76	19983.28	12165.52	2432.24	0.24	0.19
SAM: SAH	1.19	26.40	6.04	5.37	1.72	13.85	4.22	3.22	0.24	0.19
Cystine	65.23	4490.81	1427.79	1559.95	30.12	7224.91	2382.01	2031.25	0.24	0.19
SAH	3.85	49.85	24.25	14.37	6.74	47.72	28.99	11.34	0.23	0.19
Homocystine	1.74	7.59	5.01	1.51	1.28	9.15	5.76	1.74	0.22	0.19
Total cysteine	100.18	4721.59	1555.42	1660.72	52.49	7582.58	2472.32	2096.19	0.20	0.22
Total homocysteine	3.12	7.65	5.26	1.21	1.96	9.19	5.83	1.68	0.20	0.22
Riboflavin (Vit B2)	1.46	44.18	17.83	11.56	2.90	50.76	14.07	10.57	0.18	0.27
Cystathionine	13.13	55.89	32.19	9.69	20.99	104.86	39.33	18.80	0.17	0.28
Pyridoxine (Vit B6)	0.01	2.94	2.08	0.87	0.02	3.30	2.34	0.70	0.17	0.28
Glycine	8051.39	19340.24	13088.82	2691.61	7641.23	17923.68	12235.90	2589.21	0.15	0.30
Homocysteine	0.02	1.91	0.25	0.52	0.02	0.68	0.07	0.13	0.15	0.30
Homocysteic acid	0.29	24.83	4.83	5.79	0.29	24.27	7.18	6.88	0.14	0.30
Cysteine	16.26	339.26	127.62	114.16	21.32	373.08	90.30	94.95	0.10	0.44
Taurine	5420.11	28443.29	12864.73	5052.13	5939.48	21584.94	11807.59	4385.10	0.08	0.48
SAM	58.12	123.77	93.74	18.38	57.35	113.55	91.90	13.75	0.03	0.70
5-MTHFA	0.19	116.73	17.09	29.26	0.17	176.12	15.82	42.01	0.02	0.71

*ES r < 0.3: small effect; between 0.3 and 0.5: medium effect; > 0.5: large effect.*

### Conversion of betaine into DMG correlates with methylation of catechol estrogens in COC users

**Fig. 3:**
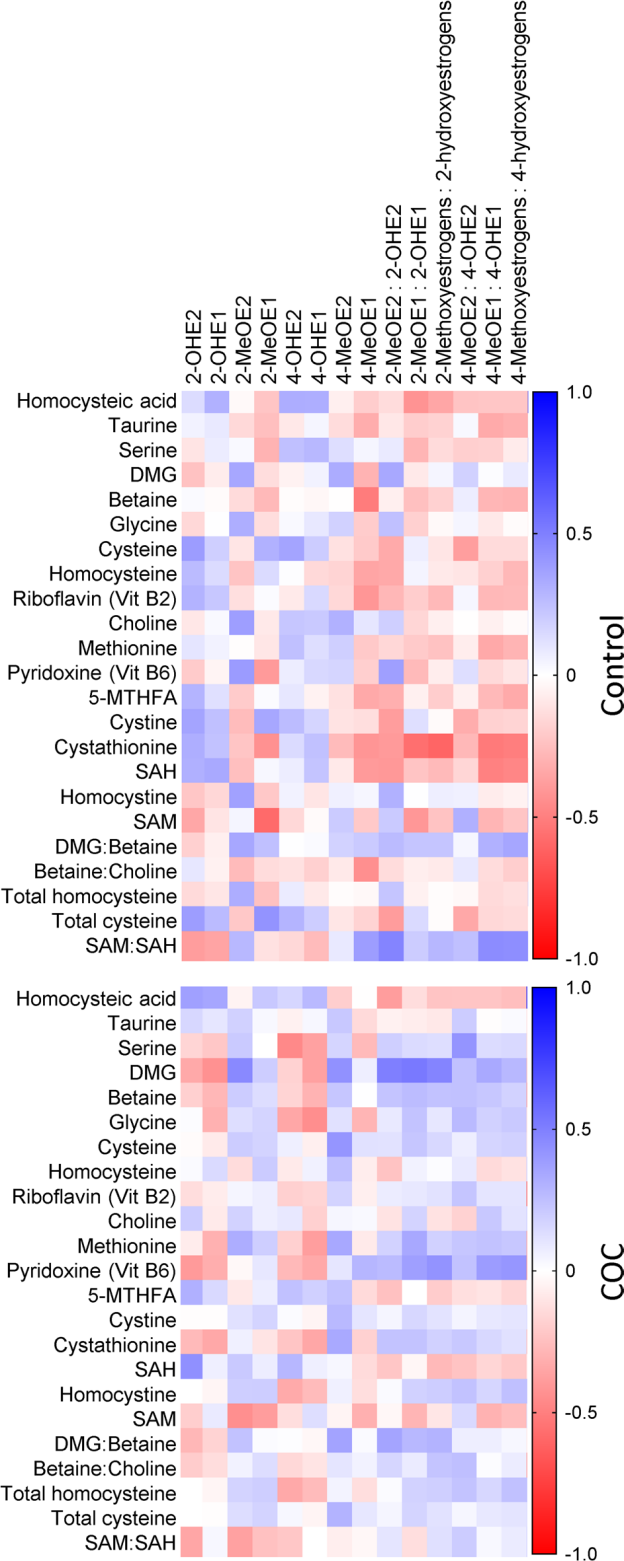
Spearman correlations between metabolites and intermediates of the methylation cycle and catechol and methoxyestrogens. Correlation analyses were performed for each experimental group separately. Spearman r values for each correlation and the associated *p* values are given in Supplementary Tables S5, S6, S7, and S8.

Spearman correlation analysis was performed to determine whether there was a correlation between the levels of metabolites and intermediates of the methylation cycle and the levels of catechol (hydroxy-) and methoxyestrogens. In the control group, 13 significant negative correlations and four positive correlations were found. Cystathionine was negatively correlated with six estrogen metabolite variables, which included 2- and 4-MeOE1 as well as four of the six methoxyestrogen: hydroxyestrogen ratios (Fig. 3 and Supplementary Tables S5 and S6). Four other methylation cycle variables negatively correlated with single estrogen metabolite variables, including betaine, riboflavin (Vit B2), SAM, and the betaine: choline ratio, while total cysteine positively correlated with 2-MeOE1. In the COC user group, on the other hand, a total of 14 significant correlations were found, of which most (10) were positive (Fig. 3 and Supplementary Tables S7 and S8). Nine of the positive correlations were between serine, DMG, cysteine, or pyridoxine (Vit B6) and the methoxyestrogens or methoxyestrogen: hydroxyestrogen ratios with DMG involved in five of these. The negative correlations in the COC user group were found between serine and 4-OHE2, DMG and 2-OHE1, glycine and 4-OHE1, as well as SAM and 2-MeOE2. Interestingly, the strong negative correlations found for cystathionine in the control group were completely lost in the COC user group (Fig. 3). These results suggest that there is an increased production of DMG from betaine to feed the increased consumption of SAM which drives the increased methylation of catechol estrogens in the COC users.

## Discussion

Biotransformation reactions in the liver play a vital role in the removal of potentially harmful metabolites by producing more water-soluble and excretable end products. These reactions are under the control of several biotransformation enzymes which are sensitive to factors such as cofactor and conjugation substrate depletion and the inhibition (and sometimes the upregulation) of specific enzymes in the system. Estrogens play a key role in the regulation of multiple physiological processes, such as sexual development, the stress response, tissue differentiation and the regulation of energy metabolism. The concentration and type of estrogen in circulation vary substantially over the lifespan of women. Although estrogens have key biological roles, the effective biotransformation and excretion of estrogens is equally important. This is highlighted by the findings of other studies suggesting that increased urinary excretion of parent estrogens and certain metabolites (e.g. 2-OHE2) results in a decreased cancer risk, whereas increased excretion of other metabolites (e.g., 16-hydroxylation metabolites and catechol estrogen DNA adducts) is associated with increased cancer risk^10,28,29^. Here, we have investigated the effect of combined oral contraceptives containing DRSP and EE on the endogenous hormone biotransformation profile in pre-menopausal women.

Although E2 is the principal estrogen in the bloodstream in premenopausal women, E1 and its metabolites had the highest abundance in urine of both the control and COC users (Table 2). This is in accordance with previous studies^30,31^. The log *P* value of E2 is 4.0 in comparison to that of estrone at 3.1, which indicates that E1 is more water soluble than E2. This is due to the presence of the ketone group in E1 which is replaced by a less polar OH group in E2. Therefore, it seems that E2 is converted into E1 and its metabolites for excretion. This is supported by a stronger association between unconjugated serum E2 and urinary E1 than between serum and urinary E2^31^ and may be a mechanism to regulate estrogen exposure, since E1 is considered to be estrogenically less active than E2^32^.

Our data further clearly showed that COC use reduced urinary free estrogen levels (E1 and E2) but not total hormone excretion. The estrogen precursors progesterone and androstenedione also tended to be lower although the reduction was not significant. These findings may suggest an inhibitory effect of COCs on the biosynthesis of endogenous steroid hormones. In line with these findings, Louw-du Toit et al.^33^ reported that the progestin component of the COCs used in this study (DRSP) inhibited the activity of both 3β-hydroxysteroid dehydrogenase 2 (3βHSD2) and cytochrome P450 17α-hydroxylase/17,20 lyase (CYP17A1) in COS cells transfected with the genes that encode these enzymes. CYP17A1 and 3βHSD2 are involved in the conversion of pregnenolone into progesterone and then to androstenedione. 3βHSD2 is also responsible for the conversion of E2 into E1. The inhibitory effect of DRSP on 3βHSD2 may, therefore, be partly responsible for the marked reduction in estrone levels in COC users.

An interesting observation, however, was that the catechol estrogen [2-OHE2(E1) and 4-OHE2(E1)] levels tended to be higher even though the E1 and E2 levels were significantly lower in the COC group. With respect to the methylation of these catechols, 2- and 4-MeOE2 and 4-MeOE1 levels were unchanged, while 2-MeOE1 was significantly increased in COC users. Importantly, estrogen DNA-adduct formation was not increased, but rather decreased in COC users. In addition, the glutathione conjugates 2&4-OHE2-Cys, 2&4-OHE1-SG, and 2&4-OHE2-SG were significantly decreased, whereas 2&4-OHE2(E1)-NAcCys and 2&4-OHE1-Cys levels were unaffected in COC users. The sulphate and glucuronide conjugate levels were also not significantly affected. This finding suggested that a larger fraction of the available parent estrogens in COC users is converted into catechol estrogens and, especially in the case of E1, to methoxyestrogens. Since the total hormone metabolite excretion was not decreased in the COC users, we calculated certain metabolite ratios and metabolite fractions to determine whether the flux through the biotransformation pathway was altered by COC use. Most metabolite ratios were increased in the COC group. The ratios affected most significantly were those between E1 and its metabolites and included 2- and 4-MeOE1:E1, 2- and 4-OHE1:E1, as well as E1-3-sulphate : E1, E1-3-glucuronide : E1. This may partly be because estrone itself was markedly decreased in COC users. Interestingly, however, the ratios between E2 and its metabolites were not significantly affected by COC use, even though 17β-estradiol levels were also significantly reduced in COC users. These findings may indicate that the conversion rate of E1 into its conjugated or hydroxylated and methylated metabolites is increased in COC users which may have contributed to the reduction in free estrogen levels that we have observed here, and which others have observed in circulation. It is uncertain to what extend EE could have contributed to the pool of hydroxylated estrogens. In vivo and in vitro studies have reported that EE itself is biotransformed for excretion mainly by glucuronidation (85%) and sulfation (12%) and that hydroxylation (mainly by CYP3A4 and CYP2A9 on C2) occurs only at low levels^22^. An alternative oxidative pathway for EE that has been identified is removal of the ethinyl group (from EE or its metabolites), which then renders E1 or E2 metabolites, with significant formation of 2-hydroxyestrogens^34^. However, only approximately 15% of the EE is excreted as de-ethynylated metabolites. Furthermore, EE and its metabolites seem to be excreted mainly in bile^35^. De-ethynylation of EE may, therefore, have contributed to the increased formation of 2-OH catechol estrogens and their methylation, although any contribution of this pathway would most likely have been small.

It appears, that only a small fraction of the catechol estrogens in the COC users were converted into estrogen-DNA adducts or glutathione conjugates, since the overall abundance of these were either decreased or were unaffected (Tables 1 and 2; Fig. 1). In further support of this, Spearman correlation analysis indicated that although there was no significant correlation between the 2- and 4-hydroxyestrogens and the 4-hydroxyestrogen DNA-adducts or glutathione conjugates, the nature of the relationship between these metabolites changed from positive in the control women to negative in the COC users (Fig. 2). Thus, there seems to be a net accumulation of, or relative increase in, hydroxylated (especially 2-hydroxylated) estrogens in COC users. 2-OHE1 and 2-OHE2 have lower estrogenic activity than E1 and E2 and have been shown to have inhibitory effects on cell growth and proliferation^36^. However, it may also induce oxidative DNA damage when it undergoes redox cycling^37^. According to Yager and Davidson^38^ the 2-hydroxylation pathway results in the formation of ROS and the formation of a low level of depurinating adducts. This is because of the low reactivity of E1(E2)-2,3-quinone, which is rather involved in redox cycling than in reactions with DNA. This would be in line with the increased levels of oxidative stress that we have observed in COC users^20^. In contrast, the 4-hydroxylation pathway and the resulting 4-OH(E2/E1) quinones cause the formation of much greater levels of depurinating adenine and guanine adducts. This pathway was, however, not upregulated in COC users. Although previous studies have identified an association between COC use and a small increase in breast cancer risk^39,40^ our results suggest that the mechanisms mediating this may not involve depurinating estrogen DNA-adduct formation.

The increased formation of catechol estrogens, however, does not seem to be accompanied by a proportional increase in their methylation. Spearman correlation analyses showed that the strong positive correlation between 2-hydroxyestrogens and 2-methoxyestrogens was lost in the COC user group, while the correlation between both these metabolites and the 2-OH-DNA-adducts became significantly stronger. This may indicate that the maximum capacity to methylate the catechol estrogens had been reached, resulting in some molecules to be channelled towards quinone and adduct formation. Both betaine and DMG (serving as methyl-group donors) were present at lower levels in the COC user group. After betaine donates a methyl group to homocysteine, it forms DMG, which is further used as a methyl donor elsewhere. The decreased levels of DMG could be ascribed to the decreased levels of betaine because DMG is produced from betaine (Supplementary Fig. S2). To determine how the production and utilisation of betaine may be affected, the betaine: choline and DMG: betaine ratios were also investigated. The DMG: betaine ratio was higher in the COC group than in the control group. The decreased levels of betaine and betaine: choline ratio and increased DMG: betaine ratio in the COC user group indicate upregulation of the methylation cycle and are consistent with the results obtained by Rios-Avila et al.^41^. In addition, we found a significant increase in choline in COC users compared with controls. The increased levels of choline in COC users may be the result of increased synthesis via phosphatidylethanolamine N-methyltransferase (PEMT), since estrogen treatment has been found to increase the activity of PEMT, leading to increased synthesis of choline^42^. However, despite this increase in its precursor, betaine levels seemed to have been insufficient to meet the increased demand for methyl-group donors in COC users. DMG levels in the COC user group were positively correlated with methoxyestrogens but negatively correlated with hydroxyestrogens, which may indicate that the methylation of estrogens is dependent on the methylation capacity provided by the betaine-DMG conversion. Although methionine levels tended to be higher in the COC user group, betaine and DMG levels were lower. This reduction in methyl group donor availability may, therefore, have resulted in insufficient methylation of estrogens and accumulation of hydroxylated metabolites. The limited availability of betaine may also have resulted in increased conversion of homocysteine to cystathionine instead of it being recycled back to methionine (Supplementary Fig. S2), since the negative correlation between cystathionine and the methylated estrogens were lost in the COC users. An alternative explanation may be that cystathionine was channeled towards glutathione synthesis to combat the oxidative stress that is induced by COC use^20^. In addition to estrogen biotransformation, the limited availability of methyl-group donors may also affect other cellular methylation reactions, e.g. DNA and histone methylation. Insufficient DNA methylation may impact fertility and embryonic development^43,44^ healthy ageing^45^ as well as carcinogenesis through the hypomethylation of proto-oncogenes and growth factors^46^. Whether COC-use may impact these (patho)physiological processes via disturbances in the methylation metabolism or redox regulation have to be further investigated.

## Conclusions

In summary, our results show that the use of COCs containing EE and DRSP causes shifts in endogenous estrogen biotransformation that result in a relative increase in the rate of estrogen conjugation, hydroxylation, and methylation, especially for E1. The increased oxidation of estrogens does not seem to result in increased estrogen DNA-adduct formation, suggesting that these molecules do not mediate the carcinogenic effect of COCs. In addition, our results indicate that there is an altered flux through the methylation cycle in COC users, which may be due to the increased demand for methyl-group donors that drive the COMT-mediated methylation of E2 and E1. The increase in estrogen methylation, however, appears to be limited by the reduced availability of methyl group donors in COC users, resulting in a tendency of especially 2-hydroxylated estrogens to accumulate.

Our study provides a comprehensive profile of 39 different urinary estrogen metabolites and precursors and 20 methionine-homocysteine and folate cycle metabolites in premenopausal women. Furthermore, this is the first study reporting the effects of COCs on the endogenous estrogen biotransformation profile. The small sample size of our study is a drawback, and the reliance on self-reported measures of health and menstrual cycle length data may have introduced biases that may have affected the robustness of our findings. Larger studies are needed to confirm these results and explore the mechanisms underlying the observed associations. Nonetheless, the innovative application of estrogen biotransformation profiling in this context represents a considerable methodological advancement through the utilisation of high-resolution LC‒MS/MS. Our findings may inform further investigations into the biological effects of COC use and the potential associated health risks, including breast carcinogenesis and effects on fertility.

## Supplementary Information

Below is the link to the electronic supplementary material.

Supplementary Material 1

## Data Availability

The datasets supporting the conclusions of this article are included within the article and its supplementary files. The raw datasets used and/or analysed during the current study are available from the corresponding author upon reasonable request.
