# Supplementary material for: Oral contraceptives containing ethinyl estradiol and drospirenone increase hydroxylation and methylation of endogenous estrogen but not genotoxic estrogen DNA-adduct formation
Source: Sci Rep. 2025 Aug 26;15:31468. doi: 10.1038/s41598-025-16892-8 (PMC12381094; doi:10.1038/s41598-025-16892-8)
Supplement: Supplementary file 1 — Supplementary Material 1 [file 41598_2025_16892_MOESM1_ESM.pdf]

Oral contraceptives containing ethinyl estradiol and drospirenone increase hydroxylation and methylation of endogenous estrogen but not genotoxic estrogen DNA-adduct formation

Gerda Venter[1,2], Carien L. van der Berg[1], Tarien Jacobs[1], Francois H. van der Westhuizen[1], and Elardus Erasmus[1].

[1]*Biomedical and Molecular Metabolism Research, Faculty of Natural and Agricultural Sciences, North-West University (Potchefstroom Campus), Potchefstroom, South Africa*

[2]*Department of Biochemistry, Faculty of Natural and Agricultural Sciences, North-West University, Mahikeng Campus, Mmabatho, South Africa*

Supplementary Material and Methods

Sample preparation and LC-MS/MS analyses of serum methylation cycle metabolites

Reagents

Deuterium labelled stable isotopes were used for internal standards. The following isotopes were purchased from Cambridge Isotopes (Tewksbury, United States): d4-L-cystine, d8-DL-homocystine, methyl-d3-methionine, d2-glycine, and d5-phenylalanine. Ion-pairing agent perfluoroheptanoic acid (PFHpA) and formic acid (FA) were purchased from Sigma Aldrich now Merck KGaA (Darmstadt, Germany). Methanol, high purity water, and acetonitrile (ACN) were purchased from Honeywell (North-Carolina, United States).

Sample preparation

The sample preparation before LC-MS/MS analysis consisted of protein precipitation, dilution, and the addition of an isotope mixture. During this sample preparation, 600 µl ACN with 1 % FA were added to 200 µl of serum for the precipitation of proteins present in the sample. After 50 µl of an internal standard stock solution was added, the mixture was vortexed. After efficient mixing, the samples were centrifuged at 4°C for 15 min at a speed of 9112 × g causing the precipitated proteins to accumulate at the bottom of the microcentrifuge tubes. The supernatant was transferred to 0.2 µm Nylon Spin-X filters. 650 µl of the supernatant were diluted with 1 ml H_2O and vortexed to ensure efficient mixing. The prepared sample was then transferred to vials for LC-MS/MS analysis.

Liquid chromatography mass spectrometry analyses

For LC-MS/MS analysis, an Agilent 1290 infinity series high pressure liquid chromatography system was used, with a vacuum degasser (G1330B), a binary pump (G4220A), temperature-controlled column compartment (G1216C) and a temperature controlled autosampler (G4226A). This LC system was coupled to an Agilent 6460 triple quadruple mass spectrometer (G6460A), with an Agilent Jet Stream ESI (AJSESI) source. These Agilent instruments were manufactured by Agilent Technologies, Santa Clara CA, USA. Liquid

chromatography separation for the metabolites and co-factors of the methionine homocysteine cycle was done by injecting 10 µl of the prepared sample onto the XSelect HSS T3 column 2,5 micron 2,1 × 100 mm (purchased from Waters). The column temperature was 30°C and mobile phase A consisted of 5 mM PFHpA in water with 0.1% formic acid and mobile phase B consisted of 100% acetonitrile with 0.1% formic acid. The mobile phase flow rate was 0.4 ml/min, and a mobile phase gradient was used. The mobile phase gradient started at 5% B and was kept isocratic for 1.5 min after which it increased to 15% in 0.5 min. It was further increased to 38% B in 2 min and to 47% B in 1 min. From 47% B it was increased to 95% in 3 min and kept isocratic for 8 min. The percentage mobile phase B was decreased to 5% in 1 min and kept isocratic for 4 min. The post run time was 2 min resulting in a final run time of 23 min. Dynamic MRM was done (see Table S1 for a summary of the ionization parameters) and an EMV of 100 V, and CAV of 4 V were used. The gas temperature was set to 250°C and the gas flow was set to 6 l/min. A nebulizer pressure of 50 psi, sheath gas temperature of 300°C, and sheath gas flow of 10 l/min were used. The capillary and nozzle voltages were set to 3000 V and 0 V respectively.

Table S1. The ionisation parameters that were used for dMRM during LC-MS/MS analysis

Analyte	Polarity	Precursor (m/z)	Product (m/z)	Fragmentor voltage (V)	Collision energy (eV)	Retention Time (min)
**L-Homocystine**	+	269.07	136	89	4	6.73
**DL-Homocysteine**	+	136.05	90	60	16	4.24
**L-Methionine**	+	150.06	104.1	74	8	5.29
**L-Cysteine**	+	122.03	59	50	24	1.90
**L-Cystine**	+	241.03	151.1	94	8	6.04
**SAM**	+	399.15	250.1	50	12	7.07
**SAH**	+	385.13	134	70	16	6.72
**Choline**	+	104.12	60.1	70	16	5.00
**Betaine**	+	118.08	58.1	118	32	1.84
**Cystathionine**	+	223.08	134	79	8	6.34
**N, N-DMG**	+	104.07	58.1	79	12	1.67
**Glycine**	+	76.04	30.1	45	8	1.85
**L-Serine**	+	106.05	60.1	50	8	1.43
**Taurine**	+	126.02	108	89	8	0.72
**L-Homocysteic acid**	+	184.03	56.1	79	20	0.70
**5-Methyltetrahydro folic acid**	+	460.2	313.1	112	16	5.78
**Riboflavin (Vitamin B_2)**	+	377.15	243.1	137	24	4.34
**Pyridoxine (Vitamin B_6)**	+	170.08	152	89	12	5.53
**d_5-Phenylalanine**	+	171.12	125.1	55	12	6.09
**d_2-Glycine**	+	78.05	32.1	35	8	1.85
**d_4-L-Cystine**	+	245.06	154	84	8	6.06
**Methyl-d_3-Methionine**	+	153.08	107	75	4	5.27
**d_8-DL-Homocystine**	+	277.12	140	79	4	6.75

Supplementary Results

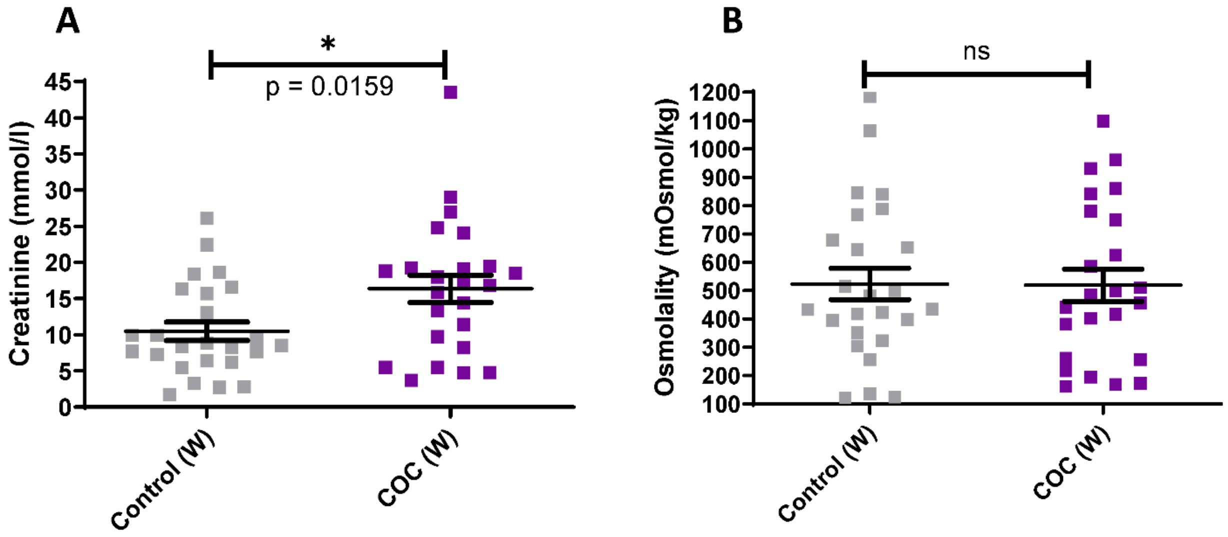

Figure S1. Urinary creatinine and osmolality measurements. *(A) Urinary creatinine concentrations of all the urine samples collected were measured at the Potchefstroom Laboratory for Inborn Errors of Metabolism (PLIEM, North-West University, South Africa) with the Thermo Scientific Konelab 20 Clinical Chemistry Analyser in mmol/L. (B) Urine osmolality was measured at Ampath Laboratories, South Africa, by determining the freezing-point of each sample using an Advanced Osmometer (model 3320). Data was analysed using the Mann-Whitney test (ns, not significant).*

Table S2. Estrogen metabolite abundances.

Variable (% of total)	Control (n=23)					COC (n=24)						
	**Min**	**Max**	**Mean**	**Std. Dev.**	**Ranking order**	**Min**	**Max**	**Mean**	**Std. Dev.**	**Ranking order**	**Mann-Whitney Effect size**	**BKY-FDR p-value**
E1-3-glucuronide	0,823	94,475	**28,551**	26,815	1	0,423	96,897	**34,024**	34,524	1	0,02	0,922
2&4-OHE2-NAcCys	0,058	60,308	**17,121**	16,786	2	0,073	63,164	**15,806**	19,721	2	0,16	0,537
2-OHE2	0,040	21,675	**4,271**	5,763	8	0,019	63,904	**13,775**	20,038	3	0,11	0,704
2&4-OHE1-NAcCys	0,027	21,325	**5,679**	6,667	7	0,006	28,225	**6,763**	8,761	4	0,06	0,840
4OHE1-1-N7Gua	0,009	58,502	**11,116**	15,862	3	0,009	53,472	**6,265**	13,449	5	0,21	0,354
2&4-OHE2-Cys	0,840	16,764	**6,090**	4,076	5	0,078	36,526	**5,060**	7,757	6	**0,31**	0,177
E3-16-glucuronide	0,151	28,829	**7,395**	7,143	4	0,299	10,488	**4,321**	3,310	7	0,17	0,475
2-OHE1	0,016	3,906	**0,822**	1,138	15	0,003	22,843	**2,267**	4,765	8	0,10	0,728
4OHE2-1-N7Gua	0,045	45,335	**5,786**	9,591	6	0,017	19,647	**2,150**	4,462	9	0,29	0,182
2&4-OHE2-SG	0,056	8,070	**1,618**	1,968	11	0,006	18,456	**1,838**	4,167	10	0,24	0,321
4-OHE1	0,010	2,181	**0,450**	0,570	18	0,002	9,155	**0,923**	1,922	11	<0,01	0,972
2&4-OHE1-SG	0,037	14,168	**2,242**	2,969	9	0,033	5,155	**0,762**	1,032	12	**0,40**	0,066
E1	0,161	11,225	**2,181**	2,587	10	0,010	8,396	**0,752**	1,695	13	**0,54**	**0,006**
2&4-OHE1-Cys	0,029	10,988	**0,829**	2,233	14	0,009	3,727	**0,670**	0,870	14	0,06	0,840
2OHE1-6-N3Ade	0,011	5,230	**0,978**	1,348	13	0,018	4,876	**0,575**	1,040	15	0,25	0,263
4-OHE2	0,002	0,920	**0,261**	0,280	23	0,001	2,765	**0,525**	0,715	16	0,05	0,874
E1-3-sulphate	0,006	4,019	**0,422**	0,845	19	0,003	2,951	**0,478**	0,758	17	0,06	0,840
E3-3-sulphate	0,011	7,720	**1,032**	1,867	12	0,001	2,620	**0,389**	0,732	18	**0,42**	0,052
Androstenedione	0,037	5,671	**0,573**	1,147	17	0,008	3,147	**0,388**	0,632	19	0,14	0,577
17-Epiestriol	0,002	1,982	**0,279**	0,540	21	0,000	5,757	**0,341**	1,164	20	0,08	0,824
E2-3-glucuronide	0,009	1,319	**0,232**	0,292	24	0,002	2,081	**0,316**	0,549	21	0,06	0,840
E2-3-sulphate	0,002	7,657	**0,581**	1,616	16	0,005	0,991	**0,267**	0,291	22	0,07	0,824
4-MeOE1	0,001	2,276	**0,144**	0,468	26	0,000	1,540	**0,254**	0,494	23	0,10	0,728
Progesterone	0,011	1,570	**0,277**	0,351	22	0,001	0,830	**0,240**	0,224	24	0,02	0,961
Testosterone	0,003	7,077	**0,376**	1,462	20	0,001	0,647	**0,137**	0,173	25	0,03	0,919
2OHE2-6-N3Ade	0,003	1,695	**0,231**	0,368	25	0,003	0,573	**0,136**	0,160	26	0,06	0,840
E3	0,001	0,400	**0,078**	0,104	27	0,000	1,957	**0,102**	0,396	27	0,29	0,182
16-Ketoestradiol	0,003	0,296	**0,049**	0,081	30	0,000	1,659	**0,096**	0,338	28	0,22	0,354
16-OHE1	0,002	0,643	**0,068**	0,147	28	0,000	1,653	**0,083**	0,335	29	0,22	0,354
2-MeOE1	0,000	0,303	**0,028**	0,065	35	0,000	0,211	**0,059**	0,069	30	**0,31**	0,177
E2-17-sulphate	0,000	0,337	**0,036**	0,069	33	0,001	0,757	**0,054**	0,153	31	0,08	0,824
4OHE1-1-N3Ade	0,001	0,281	**0,045**	0,060	31	0,000	0,339	**0,054**	0,103	32	0,18	0,447
16-Epiestriol	0,000	0,417	**0,063**	0,108	29	0,000	0,443	**0,044**	0,110	33	0,22	0,354
4OHE2-1-N3Ade	0,004	0,148	**0,041**	0,042	32	0,000	0,152	**0,038**	0,044	34	0,14	0,566
17β-Estradiol	0,002	0,199	**0,033**	0,044	34	0,000	0,236	**0,020**	0,047	35	**0,31**	0,177
17α-Estradiol	0,002	0,044	**0,014**	0,012	36	0,001	0,051	**0,017**	0,014	36	0,07	0,826
E1-2-hydroxy-3-methyl ether	0,000	0,017	**0,004**	0,005	37	0,000	0,024	**0,005**	0,006	37	0,01	0,972
2-MeOE2	0,000	0,021	**0,002**	0,004	39	0,000	0,014	**0,003**	0,004	38	0,01	0,962
4-MeOE2	0,000	0,014	**0,002**	0,003	38	0,000	0,007	**0,002**	0,002	39	0,09	0,752

Estrogen metabolite abundances were calculated as a percentage of the total metabolite load. The total metabolite load was calculated as the sum of all parent estrogens, and all estrogen metabolites and precursors. ES and p-values in bold indicate practical and statistically significant changes in metabolite abundances, respectively, between controls and COC users. E2: estradiol, E1: estrone, E3: estriol, MeO: methoxy, OH: hydroxy. ES r < 0.3: small effect, between 0.3 and 0.5: medium effect, > 0.5: large effect.

Table S3. P-values of Spearman correlation analyses of grouped metabolite abundances in the control group.

Control (p-values)										
	Total sulfate and glucuronide conjugates	Total 16-OH metabolites (including E3)	2-OH estrogens	2-Methoxy estrogens	4-OH estrogens	4-Methoxy estrogens	Total estrogens (E2+E1)	Total 2-OH DNA adducts	Total 4-OH DNA adducts	Total GSH Conjugates
Total sulfate and glucuronide conjugates		0,41	0,58	0,46	0,26	0,58	0,11	0,33	**<0,001**	**0,0013**
Total 16-OH metabolites (including E3)	0,41		0,95	0,46	0,67	**0,05**	0,58	0,95	0,13	0,58
2-OH estrogens	0,58	0,95		0,06	**0,00**	0,45	0,51	0,07	0,91	0,98
2-Methoxy estrogens	0,46	0,46	0,06		0,09	0,07	0,06	0,33	0,59	0,90
4-OH estrogens	0,26	0,67	**<0,0001**	0,09		0,99	0,47	0,05	0,59	0,42
4-Methoxy estrogens	0,58	**0,05**	0,45	0,07	0,99		**0,01**	0,37	0,72	0,87
Total estrogens (E2+E1)	0,11	0,58	0,51	0,06	0,47	**0,01**		0,46	**0,04**	0,53
Total 2-OH DNA adducts	0,33	0,95	0,07	0,33	0,05	0,37	0,46		0,53	0,50
Total 4-OH DNA adducts	**<0,001**	0,13	0,91	0,59	0,59	0,72	**0,04**	0,53		0,53
Total GSH Conjugates	**0,0013**	0,58	0,98	0,90	0,42	0,87	0,53	0,50	0,53	

Significant correlations ($p < 0.05$) are indicated in bold.

Table S4. P-values of Spearman correlation analyses of grouped metabolite abundances in the COC user group.

COC (p-values)										
	Total sulfate and glucuronide conjugates	Total 16-OH metabolites (including E3)	2-OH estrogens	2-Methoxy estrogens	4-OH estrogens	4-Methoxy estrogens	Total estrogens (E2+E1)	Total 2-OH DNA adducts	Total 4-OH DNA adducts	Total GSH Conjugates
Total sulfate and glucuronide conjugates		0,05	0,81	0,73	0,73	0,61	0,21	0,09	**0,01**	**<0,0001**
Total 16-OH metabolites (including E3)	0,05		**0,04**	**<0,001**	**0,01**	**0,03**	**<0,0001**	**<0,001**	0,70	0,12
2-OH estrogens	0,81	**0,04**		0,41	**<0,0001**	0,54	0,15	**0,04**	0,23	0,20
2-Methoxy estrogens	0,73	**<0,001**	0,41		0,49	**<0,001**	**0,0001**	**0,005**	0,73	0,45
4-OH estrogens	0,73	**0,01**	**<0,0001**	0,49		0,84	**0,01**	**0,04**	0,22	0,41
4-Methoxy estrogens	0,61	**0,03**	0,54	**<0,001**	0,84		**0,002**	0,06	0,26	0,13
Total estrogens (E2+E1)	0,21	**<0,0001**	0,15	**<0,001**	**0,01**	**0,002**		**<0,001**	0,86	0,17
Total 2-OH DNA adducts	0,09	**<0,001**	**0,04**	**0,0047**	**0,04**	0,06	**<0,001**		0,45	0,08
Total 4-OH DNA adducts	**0,01**	0,70	0,23	0,73	0,22	0,26	0,86	0,45		**0,004**
Total GSH Conjugates	**<0,0001**	0,12	0,20	0,45	0,41	0,13	0,17	0,08	**0,004**	

Significant correlations ($p < 0.05$) are indicated in bold.

Table S5. Spearman r values of correlation analyses between methylation cycle metabolites and estrogen metabolites in the Control group.

	Control (Spearman r)																																				
	2-hydroxyestradiol	2-hydroxyestrone	2-Methoxyestradiol	2-Methoxyestrone	4-Hydroxyestradiol	4-Hydroxyestrone	4-Methoxyestradiol	4-Methoxyestrone	2-Methoxyestradiol : 2-hydroxyestradiol	2-Methoxyestrone : 2-hydroxyestrone	2-Methoxyestrogens : 2-hydroxyestrogens	4-Methoxyestradiol : 4-hydroxyestradiol	4-Methoxyestrone : 4-hydroxyestrone	4-Methoxyestrogens : 4-hydroxyestrogens	Homocysteic acid	Taurine	Serine	DMG	Betaine	Glycine	Cysteine	Homocysteine	Ryboflavine (Vit B2)	Choline	Methionine	Pyridoxine (Vit B6)	5-MTHFA	Cystine	Cystathionine	SAH	Homocystine	SAM	DMG:Betaine	Betaine:Choline	Total homocysteine	Total cysteine	SAM:SAH
2-hydroxyestradiol	1,00	0,79	-0,12	0,32	0,77	0,68	0,05	-0,28	-0,88	-0,31	-0,61	-0,75	-0,59	-0,66	0,14	0,05	-0,11	-0,24	0,02	-0,16	0,39	0,26	0,30	-0,10	0,10	-0,21	0,29	0,35	0,31	0,31	-0,23	-0,35	-0,19	0,09	-0,15	0,38	-0,39
2-hydroxyestrone	0,79	1,00	-0,13	0,19	0,55	0,90	0,21	-0,32	-0,73	-0,59	-0,69	-0,54	-0,74	-0,74	0,31	0,08	0,07	-0,08	-0,02	0,00	0,19	0,14	0,22	0,02	0,05	-0,05	0,12	0,25	0,24	0,34	-0,16	-0,11	-0,07	-0,06	-0,10	0,26	-0,36
2-Methoxyestradiol	-0,12	-0,13	1,00	0,20	0,01	-0,05	0,38	0,05	0,51	0,27	0,28	0,19	0,28	0,30	-0,02	-0,15	0,02	0,34	-0,14	0,31	-0,10	-0,23	-0,13	0,38	-0,01	0,39	-0,21	-0,26	-0,23	-0,25	0,37	0,04	0,34	-0,27	0,32	-0,22	0,28
2-Methoxyestrone	0,32	0,19	0,20	1,00	0,32	0,15	0,14	0,36	-0,15	0,64	0,44	-0,25	0,41	0,38	-0,23	-0,25	-0,30	-0,14	-0,28	-0,13	0,31	0,14	0,01	-0,09	-0,10	-0,40	0,01	0,34	-0,43	0,03	-0,22	-0,58	0,25	-0,14	-0,25	0,42	-0,12
4-Hydroxyestradiol	0,77	0,55	0,01	0,32	1,00	0,63	0,19	0,06	-0,65	-0,15	-0,40	-0,80	-0,34	-0,38	0,32	-0,09	0,24	-0,05	-0,01	0,02	0,35	0,01	-0,08	0,22	0,25	0,07	0,09	0,26	0,14	0,07	0,05	-0,16	0,00	-0,12	0,08	0,29	-0,16
4-Hydroxyestrone	0,68	0,90	-0,05	0,15	0,63	1,00	0,27	-0,24	-0,61	-0,57	-0,63	-0,56	-0,71	-0,67	0,32	0,04	0,27	0,05	-0,03	0,09	0,20	-0,15	0,15	0,21	0,13	0,15	-0,05	0,16	0,24	0,23	-0,10	-0,02	0,02	-0,19	-0,09	0,19	-0,27
4-Methoxyestradiol	0,05	0,21	0,38	0,14	0,19	0,27	1,00	0,09	0,07	-0,07	-0,07	0,31	-0,05	-0,01	-0,07	-0,14	0,13	0,33	0,00	0,18	-0,12	-0,17	-0,15	0,31	0,19	0,16	-0,12	-0,11	-0,27	-0,09	0,06	0,20	0,18	-0,09	-0,01	-0,10	0,08
4-Methoxyestrone	-0,28	-0,32	0,05	0,36	0,06	-0,24	0,09	1,00	0,30	0,55	0,57	0,03	0,69	0,69	-0,20	-0,33	0,04	-0,30	-0,51	-0,20	-0,21	-0,35	-0,42	0,10	-0,21	-0,19	-0,34	-0,13	-0,42	-0,40	0,03	-0,21	0,21	-0,44	-0,03	-0,18	0,38
2-Methoxyestradiol : 2-hydroxyestradiol	-0,88	-0,73	0,51	-0,15	-0,65	-0,61	0,07	0,30	1,00	0,44	0,71	0,71	0,68	0,74	-0,14	-0,10	0,08	0,34	-0,07	0,24	-0,33	-0,34	-0,28	0,19	-0,16	0,37	-0,32	-0,38	-0,40	-0,41	0,30	0,21	0,26	-0,14	0,23	-0,39	0,48
2-Methoxyestrone : 2-hydroxyestrone	-0,31	-0,59	0,27	0,64	-0,15	-0,57	-0,07	0,55	0,44	1,00	0,91	0,21	0,82	0,79	-0,43	-0,20	-0,29	-0,09	-0,25	-0,19	0,06	0,04	-0,20	-0,16	-0,21	-0,27	-0,06	0,12	-0,57	-0,24	0,00	-0,41	0,23	-0,07	-0,06	0,14	0,20
2-Methoxyestrogens : 2-hydroxyestrogens	-0,61	-0,69	0,28	0,44	-0,40	-0,63	-0,07	0,57	0,71	0,91	1,00	0,45	0,89	0,90	-0,35	-0,18	-0,14	0,04	-0,18	-0,03	-0,10	-0,09	-0,28	-0,06	-0,24	-0,07	-0,20	-0,02	-0,61	-0,27	0,07	-0,24	0,23	-0,08	-0,01	-0,01	0,28
4-Methoxyestradiol : 4-hydroxyestradiol	-0,75	-0,54	0,19	-0,25	-0,80	-0,56	0,31	0,03	0,71	0,21	0,45	1,00	0,38	0,44	-0,24	0,03	-0,19	0,18	0,07	0,04	-0,38	-0,10	0,03	0,00	-0,07	0,13	-0,06	-0,32	-0,28	-0,16	0,06	0,31	0,03	0,09	-0,03	-0,34	0,24
4-Methoxyestrone : 4-hydroxyestrone	-0,59	-0,74	0,28	0,41	-0,34	-0,71	-0,05	0,69	0,68	0,82	0,89	0,38	1,00	0,98	-0,23	-0,34	-0,18	0,01	-0,29	-0,09	-0,14	-0,18	-0,28	-0,06	-0,34	-0,15	-0,28	-0,18	-0,52	-0,50	-0,08	-0,29	0,30	-0,14	-0,15	-0,16	0,45
4-Methoxyestrogens : 4-hydroxyestrogens	-0,66	-0,74	0,30	0,38	-0,38	-0,67	-0,01	0,69	0,74	0,79	0,90	0,44	0,98	1.00	-0,23	-0,31	-0,08	0,08	-0,30	-0,02	-0,14	-0,28	-0,27	-0,02	-0,30	-0,11	-0,34	-0,17	-0,51	-0,48	-0,05	-0,23	0,35	-0,19	-0,13	-0,14	0,44
Homocysteic acid	0,14	0,31	-0,02	-0,23	0,32	0,32	-0,07	-0,20	-0,14	-0,43	-0,35	-0,24	-0,23	-0,23	1,00	0,08	0,29	0,07	-0,04	-0,10	-0,02	-0,14	-0,08	0,14	-0,01	0,36	-0,04	0,00	0,03	-0,23	0,22	0,39	0,11	-0,18	0,23	-0,03	0,24
Taurine	0,05	0,08	-0,15	-0,25	-0,09	0,04	-0,14	-0,33	-0,10	-0,20	-0,18	0,03	-0,34	-0,31	0,08	1,00	-0,03	0,34	-0,02	-0,16	0,30	0,18	0,24	0,03	0,05	-0,03	0,37	0,46	0,20	0,41	0,31	0,34	0,14	-0,06	0,35	0,41	-0,30
Serine	-0,11	0,07	0,02	-0,30	0,24	0,27	0,13	0,04	0,08	-0,29	-0,14	-0,19	-0,18	-0,08	0,29	-0,03	1,00	0,38	0,20	0,57	-0,03	-0,42	-0,51	0,33	0,22	0,38	-0,43	0,04	0,12	-0,02	0,18	0,28	0,05	-0,04	0,18	0,03	0,02
DMG	-0,24	-0,08	0,34	-0,14	-0,05	0,05	0,33	-0,30	0,34	-0,09	0,04	0,18	0,01	0,08	0,07	0,34	0,38	1,00	0,21	0,58	0,18	-0,16	-0,15	0,33	0,11	0,40	-0,11	0,04	-0,02	-0,06	0,25	0,31	0,52	-0,05	0,25	0,14	0,09
Betaine	0,02	-0,02	-0,14	-0,28	-0,01	-0,03	0,00	-0,51	-0,07	-0,25	-0,18	0,07	-0,29	-0,30	-0,04	-0,02	0,20	0,21	1,00	0,54	0,02	0,40	0,11	0,15	0,43	0,19	0,15	-0,05	0,29	0,41	-0,30	0,00	-0,65	0,84	-0,27	0,01	-0,44
Glycine	-0,16	0,00	0,31	-0,13	0,02	0,09	0,18	-0,20	0,24	-0,19	-0,03	0,04	-0,09	-0,02	-0,10	-0,16	0,57	0,58	0,54	1,00	0,09	-0,06	-0,21	0,44	0,38	0,37	-0,21	-0,12	0,12	0,10	0,07	0,00	-0,01	0,24	0,11	0,00	-0,11
Cysteine	0,39	0,19	-0,10	0,31	0,35	0,20	-0,12	-0,21	-0,33	0,06	-0,10	-0,38	-0,14	-0,14	-0,02	0,30	-0,03	0,18	0,02	0,09	1,00	0,24	0,28	-0,25	0,37	-0,12	0,67	0,77	0,15	0,22	0,21	-0,28	0,13	0,15	0,25	0,85	-0,27
Homocysteine	0,26	0,14	-0,23	0,14	0,01	-0,15	-0,17	-0,35	-0,34	0,04	-0,09	-0,10	-0,18	-0,28	-0,14	0,18	-0,42	-0,16	0,40	-0,06	0,24	1,00	0,21	-0,39	0,16	-0,44	0,59	0,45	-0,11	0,47	-0,04	-0,23	-0,37	0,59	0,05	0,42	-0,43
Ryboflavine (Vit B2)	0,30	0,22	-0,13	0,01	-0,08	0,15	-0,15	-0,42	-0,28	-0,20	-0,28	0,03	-0,28	-0,27	-0,08	0,24	-0,51	-0,15	0,11	-0,21	0,28	0,21	1,00	-0,12	0,32	-0,24	0,47	0,19	0,40	0,36	-0,31	-0,10	-0,25	0,15	-0,25	0,23	-0,35
Choline	-0,10	0,02	0,38	-0,09	0,22	0,21	0,31	0,10	0,19	-0,16	-0,06	0,00	-0,06	-0,02	0,14	0,03	0,33	0,33	0,15	0,44	-0,25	-0,39	-0,12	1,00	0,25	0,47	-0,39	-0,45	0,11	-0,13	0,18	0,47	0,11	-0,34	0,12	-0,39	0,17
Methionine	0,10	0,05	-0,01	-0,10	0,25	0,13	0,19	-0,21	-0,16	-0,21	-0,24	-0,07	-0,34	-0,30	-0,01	0,05	0,22	0,11	0,43	0,38	0,37	0,16	0,32	0,25	1,00	-0,01	0,41	0,29	0,41	0,40	0,08	0,23	-0,21	0,24	0,11	0,33	-0,37
Pyridoxine (Vit B6)	-0,21	-0,05	0,39	-0,40	0,07	0,15	0,16	-0,19	0,37	-0,27	-0,07	0,13	-0,15	-0,11	0,36	-0,03	0,38	0,40	0,19	0,37	-0,12	-0,44	-0,24	0,47	-0,01	1,00	-0,15	-0,43	0,06	-0,21	0,47	0,44	0,01	-0,10	0,40	-0,40	0,25
5-MTHFA	0,29	0,12	-0,21	0,01	0,09	-0,05	-0,12	-0,34	-0,32	-0,06	-0,20	-0,06	-0,28	-0,34	-0,04	0,37	-0,43	-0,11	0,15	-0,21	0,67	0,59	0,47	-0,39	0,41	-0,15	1,00	0,59	0,17	0,33	0,28	-0,07	-0,20	0,36	0,33	0,59	-0,29
Cystine	0,35	0,25	-0,26	0,34	0,26	0,16	-0,11	-0,13	-0,38	0,12	-0,02	-0,32	-0,18	-0,17	0,00	0,46	0,04	0,04	-0,05	-0,12	0,77	0,45	0,19	-0,45	0,29	-0,43	0,59	1,00	-0,09	0,45	0,16	-0,24	0,06	0,17	0,24	0,97	-0,45
Cystathionine	0,31	0,24	-0,23	-0,43	0,14	0,24	-0,27	-0,42	-0,40	-0,57	-0,61	-0,28	-0,52	-0,51	0,03	0,20	0,12	-0,02	0,29	0,12	0,15	-0,11	0,40	0,11	0,41	0,06	0,17	-0,09	1,00	0,33	-0,24	0,22	-0,25	0,13	-0,20	-0,04	-0,35
SAH	0,31	0,34	-0,25	0,03	0,07	0,23	-0,09	-0,40	-0,41	-0,24	-0,27	-0,16	-0,50	-0,48	-0,23	0,41	-0,02	-0,06	0,41	0,10	0,22	0,47	0,36	-0,13	0,40	-0,21	0,33	0,45	0,33	1,00	-0,16	-0,04	-0,47	0,40	-0,09	0,43	-0,97
Homocystine	-0,23	-0,16	0,37	-0,22	0,05	-0,10	0,06	0,03	0,30	0,00	0,07	0,06	-0,08	-0,05	0,22	0,31	0,18	0,25	-0,30	0,07	0,21	-0,04	-0,31	0,18	0,08	0,47	0,28	0,16	-0,24	-0,16	1,00	0,45	0,36	-0,39	0,98	0,10	0,29
SAM	-0,35	-0,11	0,04	-0,58	-0,16	-0,02	0,20	-0,21	0,21	-0,41	-0,24	0,31	-0,29	-0,23	0,39	0,34	0,28	0,31	0,00	0,00	-0,28	-0,23	-0,10	0,47	0,23	0,44	-0,07	-0,24	0,22	-0,04	0,45	1.00	0,16	-0,29	0,38	-0,32	0,19
DMG:Betaine	-0,19	-0,07	0,34	0,25	0,00	0,02	0,18	0,21	0,26	0,23	0,23	0,03	0,30	0,35	0,11	0,14	0,05	0,52	-0,65	-0,01	0,13	-0,37	-0,25	0,11	-0,21	0,01	-0,20	0,06	-0,25	-0,47	0,36	0,16	1,00	-0,73	0,32	0,11	0,48
Betaine:Choline	0,09	-0,06	-0,27	-0,14	-0,12	-0,19	-0,09	-0,44	-0,14	-0,07	-0,08	0,09	-0,14	-0,19	-0,18	-0,06	-0,04	-0,05	0,84	0,24	0,15	0,59	0,15	-0,34	0,24	-0,10	0,36	0,17	0,13	0,40	-0,39	-0,29	-0,73	1,00	-0,32	0,19	-0,45
Total homocysteine	-0,15	-0,10	0,32	-0,25	0,08	-0,09	-0,01	-0,03	0,23	-0,06	-0,01	-0,03	-0,15	-0,13	0,23	0,35	0,18	0,25	-0,27	0,11	0,25	0,05	-0,25	0,12	0,11	0,40	0,33	0,24	-0,20	-0,09	0,98	0,38	0,32	-0,32	1,00	0,18	0,23
Total cysteine	0,38	0,26	-0,22	0,42	0,29	0,19	-0,10	-0,18	-0,39	0,14	-0,01	-0,34	-0,16	-0,14	-0,03	0,41	0,03	0,14	0,01	0,00	0,85	0,42	0,23	-0,39	0,33	-0,40	0,59	0,97	-0,04	0,43	0,10	-0,32	0,11	0,19	0,18	1,00	-0,46
SAM:SAH	-0,39	-0,36	0,28	-0,12	-0,16	-0,27	0,08	0,38	0,48	0,20	0,28	0,24	0,45	0,44	0,24	-0,30	0,02	0,09	-0,44	-0,11	-0,27	-0,43	-0,35	0,17	-0,37	0,25	-0,29	-0,45	-0,35	-0,97	0,29	0,19	0,48	-0,45	0,23	-0,46	1,00

Table S6. P values of Spearman correlation analyses between methylation cycle metabolites and estrogen metabolites in the Control group.

	Control (p-values)																																				
	2-hydroxyestradiol	2-hydroxyestrone	2-Methoxyestradiol	2-Methoxyestrone	4-Hydroxyestradiol	4-Hydroxyestrone	4-Methoxyestradiol	4-Methoxyestrone	2-Methoxyestradiol : 2-hydroxyestradiol	2-Methoxyestrone : 2-hydroxyestrone	2-Methoxyestrogens : 2-hydroxyestrogens	4-Methoxyestradiol : 4-hydroxyestradiol	4-Methoxyestrone : 4-hydroxyestrone	4-Methoxyestrogens : 4-hydroxyestrogens	Homocysteic acid	Taurine	Serine	DMG	Betaine	Glycine	Cysteine	Homocysteine	Ryboflavine (Vit B2)	Choline	Methionine	Pyridoxine (Vit B6)	5-MTHFA	Cystine	Cystathionine	SAH	Homocystine	SAM	DMG:Betaine	Betaine:Choline	Total homocysteine	Total cysteine	SAM:SAH
2-hydroxyestradiol		**0,000**	0,588	0,139	**0,000**	**0,000**	0,823	0,192	**0,000**	0,155	**0,002**	**0,000**	**0,004**	**0,001**	0,538	0,819	0,618	0,266	0,936	0,477	0,069	0,224	0,168	0,664	0,647	0,342	0,176	0,097	0,144	0,154	0,299	0,103	0,391	0,673	0,503	0,072	0,069
2-hydroxyestrone	**0,000**		0,564	0,376	**0,006**	**0,000**	0,347	0,133	**0,000**	**0,003**	**0,000**	**0,008**	**0,000**	**0,000**	0,157	0,707	0,737	0,730	0,932	0,989	0,394	0,529	0,321	0,922	0,819	0,833	0,587	0,256	0,272	0,117	0,468	0,622	0,768	0,799	0,647	0,222	0,092
2-Methoxyestradiol	0,588	0,564		0,367	0,955	0,807	0,070	0,825	**0,013**	0,221	0,199	0,383	0,213	0,174	0,913	0,495	0,925	0,111	0,514	0,145	0,641	0,283	0,568	0,075	0,968	0,065	0,342	0,222	0,293	0,241	0,087	0,868	0,111	0,212	0,138	0,319	0,200
2-Methoxyestrone	0,139	0,376	0,367		0,140	0,494	0,535	0,094	0,497	**0,001**	**0,035**	0,252	0,056	0,082	0,291	0,244	0,165	0,538	0,203	0,541	0,157	0,526	0,957	0,687	0,644	0,061	0,954	0,108	**0,040**	0,900	0,317	**0,003**	0,256	0,523	0,258	**0,044**	0,587
4-Hydroxyestradiol	**0,000**	**0,006**	0,955	0,140		**0,001**	0,383	0,797	**0,001**	0,503	0,062	**0,000**	0,126	0,080	0,136	0,677	0,262	0,805	0,946	0,922	0,098	0,975	0,703	0,308	0,256	0,754	0,680	0,229	0,523	0,761	0,826	0,477	0,989	0,584	0,730	0,176	0,477
4-Hydroxyestrone	**0,000**	**0,000**	0,807	0,494	**0,001**		0,208	0,280	**0,002**	**0,004**	**0,001**	**0,005**	**0,000**	**0,001**	0,143	0,865	0,205	0,830	0,876	0,687	0,364	0,497	0,503	0,335	0,562	0,491	0,805	0,455	0,264	0,295	0,634	0,922	0,932	0,394	0,687	0,378	0,215
4-Methoxyestradiol	0,823	0,347	0,070	0,535	0,383	0,208		0,680	0,764	0,764	0,750	0,155	0,809	0,962	0,764	0,526	0,565	0,130	0,982	0,419	0,590	0,435	0,485	0,157	0,391	0,463	0,590	0,609	0,218	0,693	0,802	0,359	0,422	0,693	0,946	0,654	0,707
4-Methoxyestrone	0,192	0,133	0,825	0,094	0,797	0,280	0,680		0,167	**0,006**	**0,004**	0,886	**0,000**	**0,000**	0,361	0,130	0,872	0,162	**0,012**	0,357	0,336	0,098	**0,049**	0,659	0,331	0,386	0,115	0,554	**0,048**	0,060	0,881	0,327	0,343	**0,035**	0,895	0,422	0,072
2-Methoxyestradiol : 2-hydroxyestradiol	**0,000**	**0,000**	**0,013**	0,497	**0,001**	**0,002**	0,764	0,167		**0,036**	**0,000**	**0,000**	**0,001**	**0,000**	0,529	0,660	0,710	0,115	0,768	0,262	0,118	0,115	0,188	0,396	0,466	0,078	0,140	0,070	0,059	0,054	0,159	0,347	0,226	0,523	0,301	0,066	**0,020**
2-Methoxyestrone : 2-hydroxyestrone	0,155	**0,003**	0,221	**0,001**	0,503	**0,004**	0,764	**0,006**	**0,036**		**0,000**	0,344	**0,000**	**0,000**	**0,042**	0,361	0,174	0,683	0,250	0,396	0,774	0,858	0,361	0,463	0,335	0,206	0,785	0,571	**0,005**	0,280	0,993	0,053	0,299	0,754	0,792	0,511	0,364
2-Methoxyestrogens : 2-hydroxyestrogens	**0,002**	**0,000**	0,199	**0,035**	0,062	**0,001**	0,750	**0,004**	**0,000**	**0,000**		**0,031**	**0,000**	**0,000**	0,100	0,419	0,514	0,865	0,409	0,907	0,641	0,690	0,201	0,774	0,270	0,740	0,368	0,918	**0,002**	0,211	0,768	0,274	0,301	0,703	0,957	0,946	0,198
4-Methoxyestradiol : 4-hydroxyestradiol	**0,000**	**0,008**	0,383	0,252	**0,000**	**0,005**	0,155	0,886	**0,000**	0,344	**0,031**		0,081	**0,041**	0,280	0,904	0,388	0,414	0,761	0,868	0,073	0,638	0,883	0,982	0,740	0,568	0,785	0,131	0,198	0,463	0,799	0,154	0,883	0,690	0,893	0,107	0,262
4-Methoxyestrone : 4-hydroxyestrone	**0,004**	**0,000**	0,213	0,056	0,126	**0,000**	0,809	**0,000**	**0,001**	**0,000**	**0,000**	0,081		**0,000**	0,311	0,126	0,414	0,970	0,196	0,695	0,523	0,411	0,206	0,782	0,122	0,506	0,210	0,420	**0,012**	**0,018**	0,732	0,187	0,172	0,533	0,519	0,490	**0,037**
4-Methoxyestrogens : 4-hydroxyestrogens	**0,001**	**0,000**	0,174	0,082	0,080	**0,001**	0,962	**0,000**	**0,000**	**0,000**	**0,000**	**0,041**	**0,000**		0,311	0,156	0,728	0,717	0,175	0,934	0,526	0,204	0,219	0,926	0,182	0,640	0,125	0,453	**0,016**	**0,025**	0,816	0,301	0,112	0,385	0,566	0,526	**0,040**
Homocysteic acid	0,538	0,157	0,913	0,291	0,136	0,143	0,764	0,361	0,529	**0,042**	0,100	0,280	0,311	0,311		0,713	0,174	0,740	0,847	0,650	0,914	0,526	0,733	0,517	0,954	0,094	0,868	0,989	0,893	0,293	0,308	0,069	0,606	0,409	0,284	0,876	0,280
Taurine	0,819	0,707	0,495	0,244	0,677	0,865	0,526	0,130	0,660	0,361	0,419	0,904	0,126	0,156	0,713		0,893	0,117	0,939	0,471	0,171	0,414	0,268	0,907	0,837	0,886	0,080	**0,027**	0,349	0,054	0,144	0,109	0,532	0,792	0,100	0,053	0,162
Serine	0,618	0,737	0,925	0,165	0,262	0,205	0,565	0,872	0,710	0,174	0,514	0,388	0,414	0,728	0,174	0,893		0,070	0,361	**0,004**	0,897	**0,049**	**0,013**	0,130	0,306	0,076	**0,042**	0,847	0,593	0,918	0,422	0,200	0,809	0,851	0,409	0,897	0,918
DMG	0,266	0,730	0,111	0,538	0,805	0,830	0,130	0,162	0,115	0,683	0,865	0,414	0,970	0,717	0,740	0,117	0,070		0,344	**0,004**	0,409	0,468	0,488	0,119	0,603	0,058	0,612	0,854	0,932	0,781	0,246	0,151	**0,012**	0,809	0,244	0,532	0,693
Betaine	0,936	0,932	0,514	0,203	0,946	0,876	0,982	**0,012**	0,768	0,250	0,409	0,761	0,196	0,175	0,847	0,939	0,361	0,344		**0,008**	0,925	0,061	0,606	0,508	**0,039**	0,373	0,494	0,805	0,187	0,053	0,158	0,982	**0,001**	**0,000**	0,218	0,975	**0,036**
Glycine	0,477	0,989	0,145	0,541	0,922	0,687	0,419	0,357	0,262	0,396	0,907	0,868	0,695	0,934	0,650	0,471	**0,004**	**0,004**	**0,008**		0,690	0,795	0,333	**0,036**	0,074	0,080	0,340	0,590	0,587	0,654	0,747	0,996	0,971	0,274	0,628	0,986	0,625
Cysteine	0,069	0,394	0,641	0,157	0,098	0,364	0,590	0,336	0,118	0,774	0,641	0,073	0,523	0,526	0,914	0,171	0,897	0,409	0,925	0,690		0,262	0,200	0,240	0,082	0,574	**0,000**	**0,000**	0,506	0,319	0,347	0,196	0,550	0,491	0,246	**0,000**	0,211
Homocysteine	0,224	0,529	0,283	0,526	0,975	0,497	0,435	0,098	0,115	0,858	0,690	0,638	0,411	0,204	0,526	0,414	**0,049**	0,468	0,061	0,795	0,262		0,333	0,063	0,463	**0,036**	**0,003**	**0,033**	0,618	**0,025**	0,840	0,293	0,080	**0,003**	0,816	**0,046**	**0,040**
Ryboflavine (Vit B2)	0,168	0,321	0,568	0,957	0,703	0,503	0,485	**0,049**	0,188	0,361	0,201	0,883	0,206	0,219	0,733	0,268	**0,013**	0,488	0,606	0,333	0,200	0,333		0,593	0,138	0,264	**0,023**	0,396	0,056	0,093	0,155	0,641	0,248	0,488	0,244	0,288	0,106
Choline	0,664	0,922	0,075	0,687	0,308	0,335	0,157	0,659	0,396	0,463	0,774	0,982	0,782	0,926	0,517	0,907	0,130	0,119	0,508	**0,036**	0,240	0,063	0,593		0,260	**0,023**	0,067	**0,031**	0,615	0,562	0,399	**0,025**	0,603	0,110	0,593	0,063	0,433
Methionine	0,647	0,819	0,968	0,644	0,256	0,562	0,391	0,331	0,466	0,335	0,270	0,740	0,122	0,182	0,954	0,837	0,306	0,603	**0,039**	0,074	0,082	0,463	0,138	0,260		0,946	0,053	0,173	0,054	0,061	0,707	0,297	0,340	0,262	0,609	0,121	0,078
Pyridoxine (Vit B6)	0,342	0,833	0,065	0,061	0,754	0,491	0,463	0,386	0,078	0,206	0,740	0,568	0,506	0,640	0,094	0,886	0,076	0,058	0,373	0,080	0,574	**0,036**	0,264	**0,023**	0,946		0,503	**0,041**	0,774	0,344	**0,023**	**0,037**	0,961	0,657	0,056	0,058	0,252
5-MTHFA	0,176	0,587	0,342	0,954	0,680	0,805	0,590	0,115	0,140	0,785	0,368	0,785	0,210	0,125	0,868	0,080	**0,042**	0,612	0,494	0,340	**0,000**	**0,003**	**0,023**	0,067	0,053	0,503		**0,003**	0,425	0,129	0,201	0,750	0,364	0,096	0,121	**0,003**	0,182
Cystine	0,097	0,256	0,222	0,108	0,229	0,455	0,609	0,554	0,070	0,571	0,918	0,131	0,420	0,453	0,989	**0,027**	0,847	0,854	0,805	0,590	**0,000**	**0,033**	0,396	**0,031**	0,173	**0,041**	**0,003**		0,690	**0,031**	0,466	0,262	0,788	0,425	0,268	**0,000**	**0,032**
Cystathionine	0,144	0,272	0,293	**0,040**	0,523	0,264	0,218	**0,048**	0,059	**0,005**	**0,002**	0,198	**0,012**	**0,016**	0,893	0,349	0,593	0,932	0,187	0,587	0,506	0,618	0,056	0,615	0,054	0,774	0,425	0,690		0,119	0,278	0,308	0,246	0,562	0,356	0,854	0,104
SAH	0,154	0,117	0,241	0,900	0,761	0,295	0,693	0,060	0,054	0,280	0,211	0,463	**0,018**	**0,025**	0,293	0,054	0,918	0,781	0,053	0,654	0,319	**0,025**	0,093	0,562	0,061	0,344	0,129	**0,031**	0,119		0,477	0,854	**0,024**	0,057	0,680	**0,038**	**0,000**
Homocystine	0,299	0,468	0,087	0,317	0,826	0,634	0,802	0,881	0,159	0,993	0,768	0,799	0,732	0,816	0,308	0,144	0,422	0,246	0,158	0,747	0,347	0,840	0,155	0,399	0,707	**0,023**	0,201	0,466	0,278	0,477		**0,032**	0,093	0,069	**0,000**	0,647	0,183
SAM	0,103	0,622	0,868	**0,003**	0,477	0,922	0,359	0,327	0,347	0,053	0,274	0,154	0,187	0,301	0,069	0,109	0,200	0,151	0,982	0,996	0,196	0,293	0,641	**0,025**	0,297	**0,037**	0,750	0,262	0,308	0,854	**0,032**		0,480	0,187	0,077	0,143	0,394
DMG:Betaine	0,391	0,768	0,111	0,256	0,989	0,932	0,422	0,343	0,226	0,299	0,301	0,883	0,172	0,112	0,606	0,532	0,809	**0,012**	**0,001**	0,971	0,550	0,080	0,248	0,603	0,340	0,961	0,364	0,788	0,246	**0,024**	0,093	0,480		**0,000**	0,133	0,606	**0,020**
Betaine:Choline	0,673	0,799	0,212	0,523	0,584	0,394	0,693	**0,035**	0,523	0,754	0,703	0,690	0,533	0,385	0,409	0,792	0,851	0,809	**0,000**	0,274	0,491	**0,003**	0,488	0,110	0,262	0,657	0,096	0,425	0,562	0,057	0,069	0,187	**0,000**		0,131	0,378	**0,030**
Total homocysteine	0,503	0,647	0,138	0,258	0,730	0,687	0,946	0,895	0,301	0,792	0,957	0,893	0,519	0,566	0,284	0,100	0,409	0,244	0,218	0,628	0,246	0,816	0,244	0,593	0,609	0,056	0,121	0,268	0,356	0,680	**0,000**	0,077	0,133	0,131		0,414	0,301
Total cysteine	0,072	0,222	0,319	**0,044**	0,176	0,378	0,654	0,422	0,066	0,511	0,946	0,107	0,490	0,526	0,876	0,053	0,897	0,532	0,975	0,986	**0,000**	**0,046**	0,288	0,063	0,121	0,058	**0,003**	**0,000**	0,854	**0,038**	0,647	0,143	0,606	0,378	0,414		**0,027**
SAM:SAH	0,069	0,092	0,200	0,587	0,477	0,215	0,707	0,072	**0,020**	0,364	0,198	0,262	**0,037**	**0,040**	0,280	0,162	0,918	0,693	**0,036**	0,625	0,211	**0,040**	0,106	0,433	0,078	0,252	0,182	**0,032**	0,104	**0,000**	0,183	0,394	**0,020**	**0,030**	0,301	**0,027**	

Table S7. Spearman r values of correlation analyses between methylation cycle metabolites and oestrogen metabolites in the COC user group

COC (Spearman r)

	2-hydroxyestradiol	2-hydroxyestrone	2-Methoxyestradiol	2-Methoxyestrone	4-Hydroxyestradiol	4-Hydroxyestrone	4-Methoxyestradiol	4-Methoxyestrone	2-Methoxyestradiol : 2-hydroxyestradiol	2-Methoxyestrone : 2-hydroxyestrone	2-Methoxyestrogens : 2-hydroxyestrogens	4-Methoxyestradiol : 4-hydroxyestradiol	4-Methoxyestrone : 4-hydroxyestrone	4-Methoxyestrogens : 4-hydroxyestrogens	Homocysteic acid	Taurine	Serine	DMG	Betaine	Glycine	Cysteine	Homocysteine	Ryboflavine (Vit B2)	Choline	Methionine	Pyridoxine (Vit B6)	5-MTHFA	Cystine	Cystathionine	SAH	Homocystine	SAM	DMG:Betaine	Betaine:Choline	Total homocysteine	Total cysteine	SAM:SAH
2-hydroxyestradiol	1,00	0,75	-0,06	0,13	0,80	0,64	-0,25	-0,23	-0,87	-0,47	-0,81	-0,82	-0,57	-0,66	0,37	0,15	-0,17	-0,34	-0,19	0,01	-0,02	0,02	-0,14	0,20	-0,07	-0,40	0,31	-0,01	-0,27	0,43	0,00	-0,19	-0,28	-0,20	0,00	0,00	-0,35
2-hydroxyestrone	0,75	1,00	-0,30	-0,11	0,62	0,93	-0,35	-0,43	-0,82	-0,76	-0,79	-0,60	-0,88	-0,88	0,35	0,10	-0,22	-0,43	-0,29	-0,31	-0,08	0,14	-0,08	-0,08	-0,31	-0,32	0,15	-0,01	-0,34	0,06	-0,04	0,08	-0,17	-0,14	-0,04	-0,01	0,03
2-Methoxyestradiol	-0,06	-0,30	1,00	0,48	-0,03	-0,25	0,64	0,39	0,44	0,56	0,41	0,21	0,45	0,37	-0,05	0,18	0,21	0,46	0,20	0,13	0,20	-0,14	0,04	0,18	0,32	-0,03	-0,09	0,12	0,06	0,21	0,19	-0,44	0,24	0,05	0,17	0,13	-0,37
2-Methoxyestrone	0,13	-0,11	0,48	1,00	0,01	-0,16	0,40	0,61	-0,05	0,64	0,39	0,02	0,36	0,32	0,21	0,03	0,00	0,19	0,13	0,17	0,17	0,20	0,06	0,06	0,19	0,09	0,07	0,17	-0,11	0,07	0,19	-0,39	0,01	0,13	0,19	0,17	-0,25
4-Hydroxyestradiol	0,80	0,62	-0,03	0,01	1,00	0,68	-0,12	0,01	-0,63	-0,42	-0,71	-0,93	-0,48	-0,62	0,16	-0,06	-0,47	-0,18	-0,17	-0,35	0,06	-0,09	-0,19	0,09	-0,18	-0,28	0,24	0,01	-0,23	0,28	-0,34	-0,13	0,01	-0,15	-0,34	0,03	-0,22
4-Hydroxyestrone	0,64	0,93	-0,25	-0,16	0,68	1,00	-0,22	-0,36	-0,67	-0,73	-0,73	-0,60	-0,88	-0,90	0,27	0,03	-0,37	-0,37	-0,30	-0,45	-0,07	0,05	-0,17	-0,19	-0,38	-0,34	0,18	-0,05	-0,35	0,06	-0,27	0,13	-0,03	-0,11	-0,27	-0,05	0,00
4-Methoxyestradiol	-0,25	-0,35	0,64	0,40	-0,12	-0,22	1,00	0,24	0,42	0,62	0,47	0,38	0,35	0,28	-0,19	0,21	0,16	0,43	0,22	0,11	0,41	0,25	0,17	0,03	0,34	0,10	0,24	0,27	0,34	0,03	0,06	-0,04	0,36	0,10	0,05	0,30	-0,07
4-Methoxyestrone	-0,23	-0,43	0,39	0,61	0,01	-0,36	0,24	1,00	0,31	0,63	0,51	0,01	0,68	0,63	-0,01	-0,15	-0,27	0,06	0,00	-0,29	0,12	-0,07	-0,06	0,02	-0,09	0,29	-0,15	0,10	-0,18	-0,14	-0,13	-0,31	0,02	0,05	-0,13	0,09	-0,03
2-Methoxyestradiol : 2-hydroxyestradiol	-0,87	-0,82	0,44	-0,05	-0,63	-0,67	0,42	0,31	1,00	0,58	0,82	0,72	0,68	0,72	-0,39	-0,06	0,19	0,50	0,23	0,08	0,11	-0,24	0,07	-0,11	0,17	0,26	-0,25	0,04	0,23	-0,23	0,02	-0,03	0,36	0,16	0,01	0,04	0,10
2-Methoxyestrone : 2-hydroxyestrone	-0,47	-0,76	0,56	0,64	-0,42	-0,73	0,62	0,63	0,58	1,00	0,83	0,46	0,85	0,80	-0,13	-0,07	0,14	0,52	0,26	0,23	0,22	0,05	0,09	0,17	0,34	0,37	0,00	0,16	0,23	-0,04	0,18	-0,29	0,28	0,07	0,17	0,17	-0,13
2-Methoxyestrogens : 2-hydroxyestrogens	-0,81	-0,79	0,41	0,39	-0,71	-0,73	0,47	0,51	0,82	0,83	1,00	0,76	0,77	0,84	-0,23	-0,09	0,13	0,48	0,24	0,09	0,15	0,01	0,11	-0,11	0,18	0,42	-0,20	0,11	0,18	-0,27	0,19	-0,10	0,30	0,23	0,19	0,11	0,12
4-Methoxyestradiol : 4-hydroxyestradiol	-0,82	-0,60	0,21	0,02	-0,93	-0,60	0,38	0,01	0,72	0,46	0,76	1,00	0,46	0,58	-0,23	0,20	0,42	0,24	0,24	0,27	0,06	0,09	0,23	-0,18	0,22	0,24	-0,13	0,04	0,21	-0,24	0,24	0,15	0,07	0,25	0,23	0,03	0,20
4-Methoxyestrone : 4-hydroxyestrone	-0,57	-0,88	0,45	0,36	-0,48	-0,88	0,35	0,68	0,68	0,85	0,77	0,46	1,00	0,96	-0,23	0,01	0,14	0,34	0,22	0,18	0,16	-0,15	0,10	0,22	0,24	0,39	-0,11	0,09	0,15	-0,16	0,16	-0,30	0,07	0,01	0,15	0,09	0,03
4-Methoxyestrogens : 4-hydroxyestrogens	-0,66	-0,88	0,37	0,32	-0,62	-0,90	0,28	0,63	0,72	0,80	0,84	0,58	0,96	1,00	-0,26	0,02	0,15	0,28	0,17	0,21	0,18	-0,11	0,10	0,11	0,22	0,41	-0,16	0,10	0,11	-0,21	0,25	-0,26	0,03	0,07	0,24	0,10	0,08
Homocysteic acid	0,37	0,35	-0,05	0,21	0,16	0,27	-0,19	-0,01	-0,39	-0,13	-0,23	-0,23	-0,23	-0,26	1,00	-0,12	-0,16	-0,26	-0,40	-0,14	-0,11	0,19	-0,66	0,19	-0,37	-0,39	0,03	0,14	-0,27	-0,11	0,25	0,13	-0,09	-0,40	0,26	0,14	0,17
Taurine	0,15	0,10	0,18	0,03	-0,06	0,03	0,21	-0,15	-0,06	-0,07	-0,09	0,20	0,01	0,02	-0,12	1,00	0,41	-0,13	0,04	0,17	0,46	-0,19	0,11	0,09	0,23	-0,20	0,43	0,43	-0,22	0,19	0,24	-0,31	-0,15	0,01	0,23	0,41	-0,10
Serine	-0,17	-0,22	0,21	0,00	-0,47	-0,37	0,16	-0,27	0,19	0,14	0,13	0,42	0,14	0,15	-0,16	0,41	1,00	0,33	0,31	0,47	0,07	0,02	0,32	0,37	0,60	-0,17	-0,20	0,25	0,43	0,14	0,47	-0,17	0,05	0,00	0,47	0,22	-0,12
DMG	-0,34	-0,43	0,46	0,19	-0,18	-0,37	0,43	0,06	0,50	0,52	0,48	0,24	0,34	0,28	-0,26	-0,13	0,33	1,00	0,57	0,15	0,15	-0,13	0,12	0,15	0,48	0,08	-0,06	0,15	0,49	0,07	0,07	-0,19	0,66	0,29	0,07	0,16	-0,13
Betaine	-0,19	-0,29	0,20	0,13	-0,17	-0,30	0,22	0,00	0,23	0,26	0,24	0,24	0,22	0,17	-0,40	0,04	0,31	0,57	1,00	0,34	0,10	0,03	0,53	-0,12	0,56	-0,11	0,16	-0,09	0,31	0,22	-0,22	-0,17	-0,12	0,79	-0,23	-0,09	-0,21
Glycine	0,01	-0,31	0,13	0,17	-0,35	-0,45	0,11	-0,29	0,08	0,23	0,09	0,27	0,18	0,21	-0,14	0,17	0,47	0,15	0,34	1,00	0,08	0,07	0,31	0,07	0,52	-0,10	0,18	-0,07	0,18	0,31	0,30	-0,07	-0,23	0,19	0,31	-0,06	-0,34
Cysteine	-0,02	-0,08	0,20	0,17	0,06	-0,07	0,41	0,12	0,11	0,22	0,15	0,06	0,16	0,18	-0,11	0,46	0,07	0,15	0,10	0,08	1,00	0,09	-0,16	0,26	0,31	-0,08	0,54	0,84	-0,05	-0,13	0,40	-0,32	0,17	-0,05	0,40	0,85	0,10
Homocysteine	0,02	0,14	-0,14	0,20	-0,09	0,05	0,25	-0,07	-0,24	0,05	0,01	0,09	-0,15	-0,11	0,19	-0,19	0,02	-0,13	0,03	0,07	0,09	1,00	0,14	-0,09	0,03	0,08	-0,15	0,17	0,33	-0,25	0,19	0,22	-0,08	0,09	0,21	0,19	0,30
Ryboflavine (Vit B2)	-0,14	-0,08	0,04	0,06	-0,19	-0,17	0,17	-0,06	0,07	0,09	0,11	0,23	0,10	0,10	-0,66	0,11	0,32	0,12	0,53	0,31	-0,16	0,14	1,00	-0,27	0,44	0,35	-0,11	-0,29	0,27	0,07	-0,15	-0,07	-0,23	0,51	-0,15	-0,31	-0,06
Choline	0,20	-0,08	0,18	0,06	0,09	-0,19	0,03	0,02	-0,11	0,17	-0,11	-0,18	0,22	0,11	0,19	0,09	0,37	0,15	-0,12	0,07	0,26	-0,09	-0,27	1,00	0,33	-0,04	-0,01	0,44	0,15	-0,07	0,48	-0,32	0,18	-0,64	0,48	0,45	0,01
Methionine	-0,07	-0,31	0,32	0,19	-0,18	-0,38	0,34	-0,09	0,17	0,34	0,18	0,22	0,24	0,22	-0,37	0,23	0,60	0,48	0,56	0,52	0,31	0,03	0,44	0,33	1,00	-0,03	0,07	0,23	0,53	0,38	0,27	-0,37	0,01	0,26	0,26	0,24	-0,38
Pyridoxine (Vit B6)	-0,40	-0,32	-0,03	0,09	-0,28	-0,34	0,10	0,29	0,26	0,37	0,42	0,24	0,39	0,41	-0,39	-0,20	-0,17	0,08	-0,11	-0,10	-0,08	0,08	0,35	-0,04	-0,03	1,00	-0,17	0,01	0,00	-0,23	-0,03	-0,05	0,30	-0,04	-0,03	0,01	0,17
5-MTHFA	0,31	0,15	-0,09	0,07	0,24	0,18	0,24	-0,15	-0,25	0,00	-0,20	-0,13	-0,11	-0,16	0,03	0,43	-0,20	-0,06	0,16	0,18	0,54	-0,15	-0,11	-0,01	0,07	-0,17	1,00	0,26	-0,23	0,25	-0,10	-0,06	-0,15	0,11	-0,11	0,28	-0,15
Cystine	-0,01	-0,01	0,12	0,17	0,01	-0,05	0,27	0,10	0,04	0,16	0,11	0,04	0,09	0,10	0,14	0,43	0,25	0,15	-0,09	-0,07	0,84	0,17	-0,29	0,44	0,23	0,01	0,26	1,00	-0,04	-0,21	0,53	-0,27	0,35	-0,29	0,54	1,00	0,21
Cystathionine	-0,27	-0,34	0,06	-0,11	-0,23	-0,35	0,34	-0,18	0,23	0,23	0,18	0,21	0,15	0,11	-0,27	-0,22	0,43	0,49	0,31	0,18	-0,05	0,33	0,27	0,15	0,53	0,00	-0,23	-0,04	1,00	0,04	0,13	0,21	0,26	0,10	0,14	-0,02	0,05
SAH	0,43	0,06	0,21	0,07	0,28	0,06	0,03	-0,14	-0,23	-0,04	-0,27	-0,24	-0,16	-0,21	-0,11	0,19	0,14	0,07	0,22	0,31	-0,13	-0,25	0,07	-0,07	0,38	-0,23	0,25	-0,21	0,04	1,00	-0,23	-0,21	-0,21	0,29	-0,25	-0,21	-0,92
Homocystine	0,00	-0,04	0,19	0,19	-0,34	-0,27	0,06	-0,13	0,02	0,18	0,19	0,24	0,16	0,25	0,25	0,24	0,47	0,07	-0,22	0,30	0,40	0,19	-0,15	0,48	0,27	-0,03	-0,10	0,53	0,13	-0,23	1,00	-0,19	0,09	-0,41	1,00	0,55	0,20
SAM	-0,19	0,08	-0,44	-0,39	-0,13	0,13	-0,04	-0,31	-0,03	-0,29	-0,10	0,15	-0,30	-0,26	0,13	-0,31	-0,17	-0,19	-0,17	-0,07	-0,32	0,22	-0,07	-0,32	-0,37	-0,05	-0,06	-0,27	0,21	-0,21	-0,19	1,00	0,04	0,00	-0,19	-0,27	0,41
DMG:Betaine	-0,28	-0,17	0,24	0,01	0,01	-0,03	0,36	0,02	0,36	0,28	0,30	0,07	0,07	0,03	-0,09	-0,15	0,05	0,66	-0,12	-0,23	0,17	-0,08	-0,23	0,18	0,01	0,30	-0,15	0,35	0,26	-0,21	0,09	0,04	1,00	-0,24	0,09	0,37	0,16
Betaine:Choline	-0,20	-0,14	0,05	0,13	-0,15	-0,11	0,10	0,05	0,16	0,07	0,23	0,25	0,01	0,07	-0,40	0,01	0,00	0,29	0,79	0,19	-0,05	0,09	0,51	-0,64	0,26	-0,04	0,11	-0,29	0,10	0,29	-0,41	0,00	-0,24	1,00	-0,41	-0,29	-0,25
Total homocysteine	0,00	-0,04	0,17	0,19	-0,34	-0,27	0,05	-0,13	0,01	0,17	0,19	0,23	0,15	0,24	0,26	0,23	0,47	0,07	-0,23	0,31	0,40	0,21	-0,15	0,48	0,26	-0,03	-0,11	0,54	0,14	-0,25	1,00	-0,19	0,09	-0,41	1,00	0,55	0,21
Total cysteine	0,00	-0,01	0,13	0,17	0,03	-0,05	0,30	0,09	0,04	0,17	0,11	0,03	0,09	0,10	0,14	0,41	0,22	0,16	-0,09	-0,06	0,85	0,19	-0,31	0,45	0,24	0,01	0,28	1,00	-0,02	-0,21	0,55	-0,27	0,37	-0,29	0,55	1,00	0,22
SAM:SAH	-0,35	0,03	-0,37	-0,25	-0,22	0,00	-0,07	-0,03	0,10	-0,13	0,12	0,20	0,03	0,08	0,17	-0,10	-0,12	-0,13	-0,21	-0,34	0,10	0,30	-0,06	0,01	-0,38	0,17	-0,15	0,21	0,05	-0,92	0,20	0,41	0,16	-0,25	0,21	0,22	1,00

Table S8. P values of Spearman correlation analyses between methylation cycle metabolites and oestrogen metabolites in the COC user group.

	COC (p-values)																																			
	2-hydroxyestradiol	2-hydroxyestrone	2-Methoxyestradiol	2-Methoxyestrone	4-Hydroxyestradiol	4-Hydroxyestrone	4-Methoxyestradiol	4-Methoxyestrone	2-Methoxyestradiol : 2-hydroxyestradiol	2-Methoxyestrone : 2-hydroxyestrone	2-Methoxyestrogens : 2-hydroxyestrogens	4-Methoxyestradiol : 4-hydroxyestradiol	4-Methoxyestrone : 4-hydroxyestrone	4-Methoxyestrogens : 4-hydroxyestrogens	Homocysteic acid	Taurine	Serine	DMG	Betaine	Glycine	Cysteine	Homocysteine	Ryboflavine (Vit B2)	Choline	Methionine	Pyridoxine (Vit B6)	5-MTHFA	Cystine	Cystathionine	SAH	Homocystine	SAM	DMG:Betaine	Betaine:Choline	Total homocysteine	Total cysteine	SAM:SAH
2-hydroxyestradiol		**0,000**	0,773	0,533	**0,000**	**0,001**	0,236	0,286	**0,000**	**0,021**	**0,000**	**0,000**	**0,004**	**0,000**	0,076	0,475	0,438	0,106	0,386	0,965	0,936	0,933	0,527	0,355	0,728	0,054	0,142	0,965	0,197	**0,035**	0,994	0,364	0,178	0,347	0,987	0,994	0,092
2-hydroxyestrone	**0,000**		0,159	0,622	**0,001**	**0,000**	0,091	**0,036**	**0,000**	**0,000**	**0,000**	**0,002**	**0,000**	**0,000**	0,095	0,657	0,294	**0,034**	0,177	0,145	0,695	0,501	0,716	0,698	0,142	0,130	0,496	0,968	0,099	0,778	0,846	0,710	0,414	0,527	0,850	0,965	0,897
2-Methoxyestradiol	0,773	0,159		**0,018**	0,896	0,240	**0,001**	0,058	**0,032**	**0,005**	**0,049**	0,330	**0,029**	0,076	0,823	0,407	0,331	**0,024**	0,352	0,557	0,358	0,522	0,864	0,410	0,127	0,892	0,670	0,590	0,798	0,323	0,375	**0,032**	0,265	0,815	0,421	0,542	0,075
2-Methoxyestrone	0,533	0,622	**0,018**		0,958	0,463	0,054	**0,001**	0,824	**0,001**	0,061	0,933	0,086	0,132	0,314	0,904	0,990	0,366	0,535	0,433	0,431	0,340	0,768	0,768	0,375	0,662	0,747	0,440	0,619	0,756	0,368	0,062	0,945	0,538	0,377	0,421	0,248
4-Hydroxyestradiol	**0,000**	**0,001**	0,896	0,958		**0,000**	0,565	0,955	**0,001**	**0,039**	**0,000**	**0,000**	**0,017**	**0,001**	0,458	0,790	**0,022**	0,400	0,426	0,098	0,781	0,692	0,386	0,677	0,389	0,192	0,257	0,949	0,282	0,185	0,108	0,535	0,977	0,475	0,104	0,897	0,298
4-Hydroxyestrone	**0,001**	**0,000**	0,240	0,463	**0,000**		0,296	0,083	**0,000**	**0,000**	**0,000**	**0,002**	**0,000**	**0,000**	0,195	0,897	0,076	0,074	0,153	**0,029**	0,759	0,809	0,440	0,379	0,065	0,104	0,390	0,834	0,098	0,778	0,204	0,552	0,875	0,616	0,203	0,834	0,997
4-Methoxyestradiol	0,236	0,091	**0,001**	0,054	0,565	0,296		0,260	**0,042**	**0,001**	**0,019**	0,064	0,090	0,192	0,366	0,314	0,450	**0,037**	0,300	0,593	**0,044**	0,243	0,424	0,878	0,106	0,651	0,267	0,197	0,108	0,888	0,787	0,837	0,086	0,645	0,834	0,159	0,753
4-Methoxyestrone	0,286	**0,036**	0,058	**0,001**	0,955	0,083	0,260		0,145	**0,001**	**0,010**	0,968	**0,000**	**0,001**	0,974	0,491	0,198	0,768	0,994	0,169	0,585	0,738	0,768	0,920	0,689	0,177	0,496	0,648	0,391	0,506	0,546	0,139	0,933	0,815	0,538	0,674	0,878
2-Methoxyestradiol : 2-hydroxyestradiol	**0,000**	**0,000**	**0,032**	0,824	**0,001**	**0,000**	**0,042**	0,145		**0,003**	**0,000**	**0,000**	**0,000**	**0,000**	0,063	0,793	0,384	**0,012**	0,282	0,701	0,599	0,262	0,738	0,599	0,416	0,212	0,245	0,859	0,275	0,290	0,929	0,872	0,087	0,463	0,952	0,869	0,648
2-Methoxyestrone : 2-hydroxyestrone	**0,021**	**0,000**	**0,005**	**0,001**	**0,039**	**0,000**	**0,001**	**0,001**	**0,003**		**0,000**	**0,023**	**0,000**	**0,000**	0,530	0,738	0,514	**0,009**	0,220	0,275	0,302	0,824	0,680	0,426	0,104	0,073	0,994	0,468	0,269	0,862	0,405	0,169	0,191	0,728	0,419	0,440	0,546
2-Methoxyestrogens : 2-hydroxyestrogens	**0,000**	**0,000**	**0,049**	0,061	**0,000**	**0,000**	**0,019**	**0,010**	**0,000**	**0,000**		**0,000**	**0,000**	**0,000**	0,271	0,668	0,552	**0,018**	0,262	0,665	0,483	0,965	0,599	0,596	0,400	**0,040**	0,344	0,613	0,405	0,194	0,364	0,645	0,158	0,284	0,382	0,605	0,574
4-Methoxyestradiol : 4-hydroxyestradiol	**0,000**	**0,002**	0,330	0,933	**0,000**	**0,002**	0,064	0,968	**0,000**	**0,023**	**0,000**		**0,023**	**0,003**	0,281	0,353	**0,042**	0,257	0,257	0,204	0,768	0,689	0,288	0,409	0,300	0,260	0,548	0,850	0,314	0,262	0,260	0,491	0,762	0,241	0,269	0,881	0,355
4-Methoxyestrone : 4-hydroxyestrone	**0,004**	**0,000**	**0,029**	0,086	**0,017**	**0,000**	0,090	**0,000**	**0,000**	**0,000**	**0,000**	**0,023**		**0,000**	0,281	0,958	0,527	0,109	0,310	0,402	0,453	0,488	0,645	0,312	0,262	0,063	0,617	0,680	0,480	0,448	0,463	0,148	0,762	0,958	0,473	0,680	0,907
4-Methoxyestrogens : 4-hydroxyestrogens	**0,000**	**0,000**	0,076	0,132	**0,001**	**0,000**	0,192	**0,001**	**0,000**	**0,000**	**0,000**	**0,003**	**0,000**		0,225	0,929	0,491	0,192	0,419	0,328	0,407	0,593	0,639	0,613	0,304	**0,047**	0,445	0,636	0,596	0,320	0,248	0,218	0,894	0,741	0,253	0,630	0,725
Homocysteic acid	0,076	0,095	0,823	0,314	0,458	0,195	0,366	0,974	0,063	0,530	0,271	0,281	0,281	0,225		0,565	0,458	0,220	0,053	0,514	0,610	0,362	**0,000**	0,382	0,077	0,058	0,904	0,517	0,201	0,610	0,231	0,541	0,662	0,053	0,225	0,504	0,431
Taurine	0,475	0,657	0,407	0,904	0,790	0,897	0,314	0,491	0,793	0,738	0,668	0,353	0,958	0,929	0,565		**0,044**	0,560	0,850	0,431	**0,022**	0,366	0,607	0,689	0,277	0,344	**0,038**	**0,034**	0,300	0,375	0,266	0,145	0,491	0,968	0,284	**0,045**	0,651
Serine	0,438	0,294	0,331	0,990	**0,022**	0,076	0,450	0,198	0,384	0,514	0,552	**0,042**	0,527	0,491	0,458	**0,044**		0,113	0,140	**0,022**	0,759	0,939	0,132	0,076	**0,002**	0,436	0,356	0,233	**0,034**	0,527	**0,021**	0,431	0,812	0,997	**0,021**	0,306	0,563
DMG	0,106	**0,034**	**0,024**	0,366	0,400	0,074	**0,037**	0,768	**0,012**	**0,009**	**0,018**	0,257	0,109	0,192	0,220	0,560	0,113		**0,004**	0,475	0,485	0,544	0,576	0,483	**0,017**	0,719	0,790	0,498	**0,014**	0,728	0,728	0,386	**0,000**	0,171	0,759	0,450	0,535
Betaine	0,386	0,177	0,352	0,535	0,426	0,153	0,300	0,994	0,282	0,220	0,262	0,257	0,310	0,419	0,053	0,850	0,140	**0,004**		0,101	0,636	0,875	**0,008**	0,563	**0,005**	0,610	0,446	0,674	0,136	0,304	0,294	0,438	**0,568**	**0,000**	0,281	0,660	0,314
Glycine	0,965	0,145	0,557	0,433	0,098	**0,029**	0,593	0,169	0,701	0,275	0,665	0,204	0,402	0,328	0,514	0,431	**0,022**	0,475	0,101		0,719	0,750	0,147	0,753	**0,009**	0,628	0,387	0,753	0,389	0,140	0,152	0,731	0,269	0,373	0,145	0,790	0,108
Cysteine	0,936	0,695	0,358	0,431	0,781	0,759	**0,044**	0,585	0,599	0,302	0,483	0,768	0,453	0,407	0,610	**0,022**	0,759	0,485	0,636	0,719		0,674	0,445	0,228	0,138	0,698	**0,007**	**0,000**	0,818	0,552	0,051	0,127	0,426	0,812	0,053	**0,000**	0,642
Homocysteine	0,933	0,501	0,522	0,340	0,692	0,809	0,243	0,738	0,262	0,824	0,965	0,689	0,488	0,593	0,362	0,366	0,939	0,544	0,875	0,750	0,674		0,501	0,686	0,875	0,695	0,473	0,438	0,114	0,248	0,362	0,296	0,722	0,692	0,322	0,366	0,154
Ryboflavine (Vit B2)	0,527	0,716	0,864	0,768	0,386	0,440	0,424	0,768	0,738	0,680	0,599	0,288	0,645	0,639	**0,000**	0,607	0,132	0,576	**0,008**	0,147	0,445	0,501		0,198	**0,030**	0,094	0,622	0,163	0,210	0,728	0,483	0,747	0,277	**0,010**	0,475	0,145	0,793
Choline	0,355	0,698	0,410	0,768	0,677	0,379	0,878	0,920	0,599	0,426	0,596	0,409	0,312	0,613	0,382	0,689	0,076	0,483	0,563	0,753	0,228	0,686	0,198		0,113	0,865	0,966	**0,031**	0,493	0,738	**0,017**	0,126	0,389	**0,001**	**0,018**	**0,027**	0,952
Methionine	0,728	0,142	0,127	0,375	0,389	0,065	0,106	0,689	0,416	0,104	0,400	0,300	0,262	0,304	0,077	0,277	**0,002**	**0,017**	**0,005**	**0,009**	0,138	0,875	**0,030**	0,113		0,904	0,754	0,277	**0,008**	0,065	0,207	0,072	0,971	0,212	0,218	0,260	0,064
Pyridoxine (Vit B6)	0,054	0,130	0,892	0,662	0,192	0,104	0,651	0,177	0,212	0,073	**0,040**	0,260	0,063	**0,047**	0,058	0,344	0,436	0,719	0,610	0,628	0,698	0,695	0,094	0,865	0,904		0,428	0,977	0,997	0,290	0,897	0,815	0,152	0,865	0,907	0,945	0,428
5-MTHFA	0,142	0,496	0,670	0,747	0,257	0,390	0,267	0,496	0,245	0,994	0,344	0,548	0,617	0,445	0,904	**0,038**	0,356	0,790	0,446	0,387	**0,007**	0,473	0,622	0,966	0,754	0,428		0,217	0,269	0,231	0,627	0,782	0,487	0,595	0,599	**0,193**	0,482
Cystine	0,965	0,968	0,590	0,440	0,949	0,834	0,197	0,648	0,859	0,468	0,613	0,850	0,680	0,636	0,517	**0,034**	0,233	0,498	0,674	0,753	**0,000**	0,438	0,163	**0,031**	0,277	0,977	0,217		0,869	0,332	**0,007**	0,201	0,092	0,169	**0,007**	**0,000**	0,320
Cystathionine	0,197	0,099	0,798	0,619	0,282	0,098	0,108	0,391	0,275	0,269	0,405	0,314	0,480	0,596	0,201	0,300	**0,034**	**0,014**	0,136	0,389	0,818	0,114	0,210	0,493	**0,008**	0,997	0,269	0,869		0,837	0,544	0,326	0,223	0,630	0,519	0,926	0,828
SAH	**0,035**	0,778	0,323	0,756	0,185	0,778	0,888	0,506	0,290	0,862	0,194	0,262	0,448	0,320	0,610	0,375	0,527	0,728	0,304	0,140	0,552	0,248	0,728	0,738	0,065	0,290	0,231	0,332	0,837		0,269	0,316	0,326	0,166	0,248	0,316	**0,000**
Homocystine	0,994	0,846	0,375	0,368	0,108	0,204	0,787	0,546	0,929	0,405	0,364	0,260	0,463	0,248	0,231	0,266	**0,021**	0,728	0,294	0,152	0,051	0,362	0,483	**0,017**	0,207	0,897	0,627	**0,007**	0,544	0,269		0,362	0,674	**0,047**	**0,000**	**0,005**	0,338
SAM	0,364	0,710	**0,032**	0,062	0,535	0,552	0,837	0,139	0,872	0,169	0,645	0,491	0,148	0,218	0,541	0,145	0,431	0,386	0,438	0,731	0,127	0,296	0,747	0,126	0,072	0,815	0,782	0,201	0,326	0,316	0,362		0,869	0,994	0,371	0,206	**0,047**
DMG:Betaine	0,178	0,414	0,265	0,945	0,977	0,875	0,086	0,933	0,087	0,191	0,158	0,762	0,762	0,894	0,662	0,491	0,812	**0,000**	**0,568**	0,269	0,426	0,722	0,277	0,389	0,971	0,152	0,487	0,092	0,223	0,326	0,674	0,869		**0,259**	0,677	0,077	0,463
Betaine:Choline	0,347	0,527	0,815	0,538	0,475	0,616	0,645	0,815	0,463	0,728	0,284	0,241	0,958	0,741	0,053	0,968	0,997	0,171	**0,000**	0,373	0,812	0,692	**0,010**	**0,001**	0,212	0,865	0,595	0,169	0,630	0,166	**0,047**	0,994	**0,259**		**0,044**	0,166	0,231
Total homocysteine	0,987	0,850	0,421	0,377	0,104	0,203	0,834	0,538	0,952	0,419	0,382	0,269	0,473	0,253	0,225	0,284	**0,021**	0,759	0,281	0,145	0,053	0,322	0,475	**0,018**	0,218	0,907	0,599	**0,007**	0,519	0,248	**0,000**	0,371	0,677	**0,044**		**0,005**	0,318
Total cysteine	0,994	0,965	0,542	0,421	0,897	0,834	0,159	0,674	0,869	0,440	0,605	0,881	0,680	0,630	0,504	**0,045**	0,306	0,450	0,660	0,790	**0,000**	0,366	0,145	**0,027**	0,260	0,945	**0,193**	**0,000**	0,926	0,316	**0,005**	0,206	0,077	0,166	**0,005**		0,300
SAM:SAH	0,092	0,897	0,075	0,248	0,298	0,997	0,753	0,878	0,648	0,546	0,574	0,355	0,907	0,725	0,431	0,651	0,563	0,535	0,314	0,108	0,642	0,154	0,793	0,952	0,064	0,428	0,482	0,320	0,828	**0,000**	0,338	**0,047**	0,463	0,231	0,318	0,300	

Supplementary figures

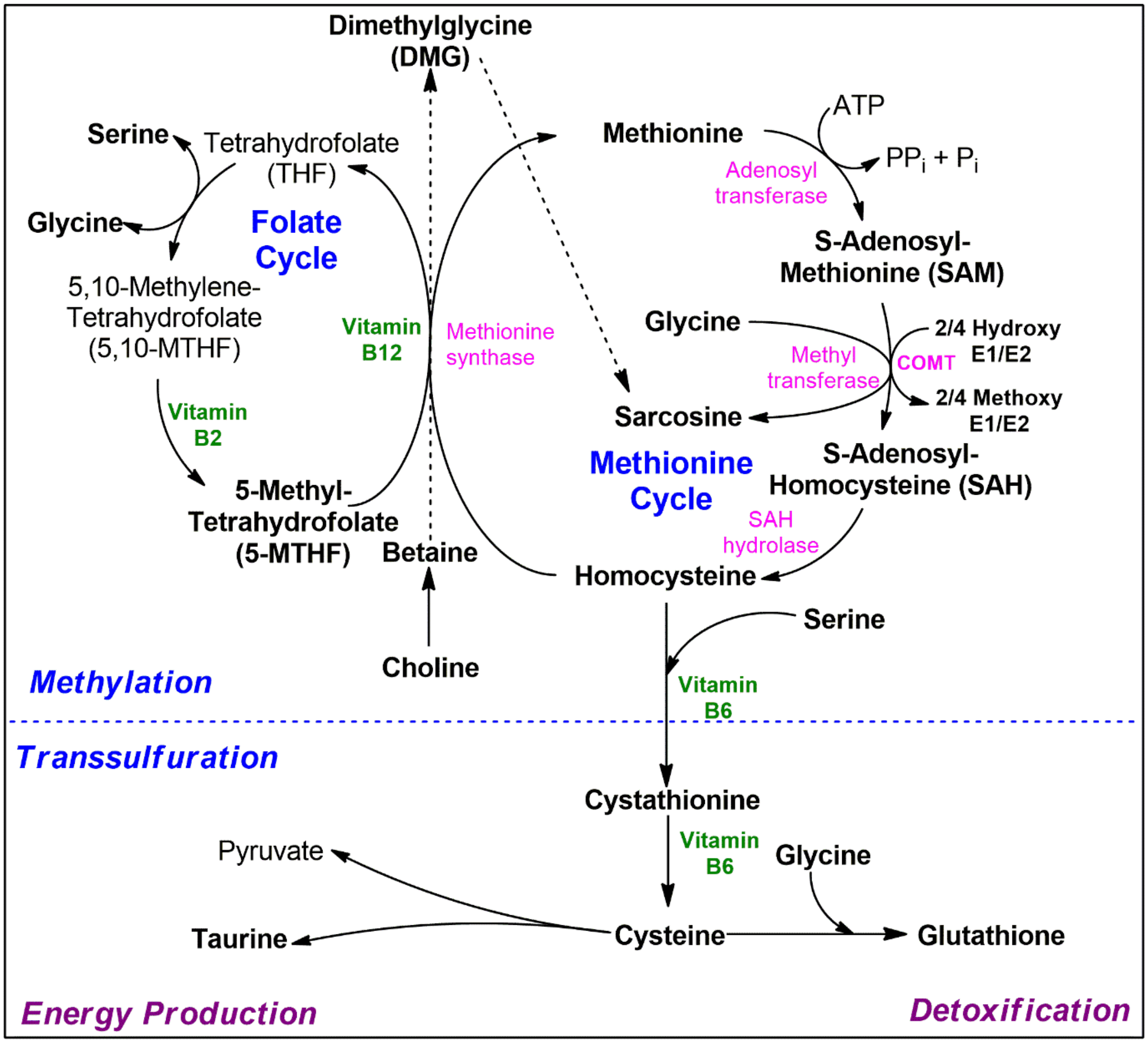

Figure S2. Schematic presentation of the methylation cycle. *The diagram indicates the most prominent co-factors, intermediates, and precursors of the methionine-homocysteine and folate cycles, as well as the transsulfuration pathway and indicates how it links to estrogen biotransformation via the COMT reaction. Modified from Gropper and Smith [1] and Guiraud et al. [2].*

References

1 Gropper, S. S., Smith, J.L. *Advanced nutrition and human metabolism*. (2012).

2 Guiraud, S. P. *et al.* High-throughput and simultaneous quantitative analysis of homocysteine-methionine cycle metabolites and co-factors in blood plasma and cerebrospinal fluid by isotope dilution LC-MS/MS. *Anal. Bioanal. Chem.* **409**, 295-305 (2017). https://doi.org:10.1007/s00216-016-0003-1
